# Enhanced MIF/CD74 axis activity shapes B cell functioning following traumatic spinal cord injury

**DOI:** 10.1186/s12974-026-03821-3

**Published:** 2026-04-22

**Authors:** Serina Rubio, Lien Beckers, Hanne Coenen, Charlotte C. M. van Laake-Geelen, Bart Depreitere, Sven Bamps, Erwin M. J. Cornips, Eveleen Buelens, Diedrik Peuskens, Jens Deckers, Veerle Somers, Judith Fraussen

**Affiliations:** 1 Department of Immunology & Infection, Biomedical Research Institute, UHasselt - Hasselt University, Hasselt, Belgium; 2 Adelante Centre of Expertise in Rehabilitation and Audiology, Hoensbroek, The Netherlands; 3 Department of Rehabilitation Medicine, Research School CAPHRI, Maastricht University, Maastricht, The Netherlands; 4 Division of Neurosurgery, University Hospitals Leuven, Leuven, Belgium; 5 Department of Neurosurgery, Jessa Hospitals, Hasselt, Belgium; 6 Department of Neurosurgery, Ziekenhuis Oost-Limburg, Genk, Belgium; 7 Department of Neurosurgery, Algemeen Ziekenhuis (AZ) Turnhout, Turnhout, Belgium

**Keywords:** B cells, Traumatic spinal cord injury, MIF/CD74 axis, High-dimensional flow cytometry, *In vitro* functional assays

## Abstract

A traumatic spinal cord injury (SCI) severely damages the nerve tissue of the spinal cord, often leading to long-term impairment of motor, sensory and autonomic functions. During a secondary injury phase, an inflammatory immune response is initiated. Current evidence points toward involvement of B cells and the macrophage migration inhibitory factor (MIF)/CD74 axis in SCI pathology. This study aimed to map the complete MIF/CD74 axis across the immune system and analyze its impact on B cell function after traumatic SCI.

Peripheral blood samples were obtained from a total cohort of 90 healthy controls (HC) and 70 SCI patients. SCI samples were collected longitudinally in the acute (0 weeks post-SCI), subacute (2 weeks post-SCI), intermediate (3–18 weeks post-SCI) and chronic (26–52 weeks post-SCI) phases post-injury. MIF levels in plasma of SCI patients and HC were measured using ELISA. Relative and absolute levels of immune and B cell subsets, as well as expression of members of the MIF/CD74 axis, were studied using high-dimensional flow cytometry. The functional relevance of MIF/CD74 axis signaling on B cell functions was investigated using *in vitro* blocking assays.

SCI triggered an early immune cell reduction, characterized by decreased absolute numbers of total and MIF/CD74 axis-expressing B cells and immune cells. In contrast, the MIF/CD74 axis was upregulated as MIF receptor surface expression on B cell subsets, as well as frequencies of CD74^+^, CD44^+^ and CXCR4^+^ B cells, were increased during the subacute/intermediate phase post-SCI. MIF plasma levels were increased in SCI patients, yet MIF expression levels were reduced within circulating immune cells, suggesting that the injured spinal cord is the main source of MIF. Importantly, decreased B cell proliferation, activation and cytokine production were observed after blocking of CD74, CD44 or MIF in primary B cells of SCI patients and HC. The observed effects were more pronounced in SCI B cells, highlighting their stronger dependence on MIF/CD74 axis signaling.

In conclusion, the complete MIF/CD74 axis plays an important role in post-SCI B cell responses. Our findings warrant further investigation of the MIF/CD74 axis as a potential target for immunomodulatory strategies in SCI treatment.

**Supplementary Information:**

The online version contains supplementary material available at 10.1186/s12974-026-03821-3.

## Introduction

Traumatic spinal cord injury (SCI) is caused by an insult to the spinal cord that severely injures the nerve tissue [1]. SCI can lead to long-term impairment of motor, sensory and autonomic functions, and these deficits can be aggravated by secondary tissue damage [2]. During this secondary injury phase, local and peripheral immune cell infiltration triggers an inflammatory immune response [2]. Within hours, neutrophils enter the lesion and are the first immune cells on site, followed by microglia and monocytes/macrophages during the first few days [3, 4]. Infiltration of lymphocytes occurs in the first weeks post-SCI [4, 5]. The involvement of B cells in SCI was indicated by their presence in the lesion of SCI mice and pigs, and in human post-mortem spinal cord tissue [6–11]. In animals, B cells already infiltrated the spinal cord lesion within the first week post-SCI, after which their numbers substantially increased over time [6–9]. In humans, B cells were primarily detected around two weeks following SCI and remained present for at least up to 36 days post-injury [10, 11]. These B cells formed ectopic follicle-like structures in the spinal cord [6, 7]. Increased antibody production was also evident post-SCI, which was mainly directed against central nervous system proteins [7, 8, 11–15]. Furthermore, B cells were shown to contribute to spinal cord pathology and impaired functional recovery as evidenced by B cell depletion and knockout mouse studies [8, 16].

Accompanying this immune cell infiltration is the production of many pro- and anti-inflammatory cytokines, such as interleukin (IL)−1β, IL-6, IL-10 and tumor necrosis factor-α (TNF-α) [2, 17]. Additionally, the cytokine macrophage migration inhibitory factor (MIF) was shown to be increased in the spinal cord of SCI animals and the peripheral blood of SCI patients [18–23]. This cytokine is highly expressed in multiple cell types (e.g., lymphocytes, monocytes/macrophages, astrocytes, endothelial cells, epithelial cells) and can bind to its surface receptor CD74, which is mainly found on antigen-presenting cells, such as B cells, dendritic cells (DCs) and macrophages, but also on other cell types under inflammatory conditions [24, 25]. MIF binding to CD74 leads to recruitment of additional receptors, namely the co-receptor CD44 or CXC-motif chemokine receptors (CXCR) 2, 4 and 7. This activates intracellular signaling pathways, such as the extracellular signal-regulated kinase (ERK)/mitogen-activated protein kinase (MAPK) pathway, that eventually promote cell survival, proliferation, migration and cytokine production [24].

In traumatic SCI, the MIF/CD74 axis has been mainly studied in astrocytes and microglia, and was shown to be involved in the regulation of chemokine expression, cholesterol metabolism, intracellular pathways (e.g., MAPK and nuclear factor κB (NF-κB) pathways) and cell recruitment to the lesion site (as reviewed in [26]) [18, 27–31]. In neurons, the MIF/CD74 axis induced oxidative stress, regulated intracellular calcium levels and reduced viability [32–34]. MIF and CD74 were also reported to be involved in recruiting lymphocytes to the lesion site [35]. Similar to B cells, genetic manipulation of MIF (inhibition or knockout) in traumatic SCI mouse models reduced spinal cord pathology and improved functional recovery [27, 28, 33, 35–37].

Current evidence thus points toward the involvement of both B cells and the MIF/CD74 axis in SCI pathology. Interestingly, we previously reported increased frequencies of circulating CD74^+^ B cells in SCI patients compared to healthy controls (HC) [38]. This raises the question whether the MIF/CD74 axis plays a role in post-SCI B cell responses. Therefore, this study aimed to map the entire MIF/CD74 axis across the immune system and analyze its impact on B cell function after SCI. We thoroughly examined plasma MIF levels and all players of the MIF/CD74 axis in the major circulating immune cell subsets, with a special focus on B cell subsets, in a longitudinal SCI patient cohort by ELISA and high-dimensional flow cytometry. In addition, the effect of interfering with MIF/CD74 axis signaling on B cell functions was studied using *in vitro* functional assays for proliferation, activation and cytokine production.

## Materials and methods

### Study subjects

SCI patients were recruited at Ziekenhuis Oost-Limburg (Genk, Belgium), University Hospitals Leuven (Leuven, Belgium), AZ Turnhout (Turnhout, Belgium), Jessa Hospitals (Hasselt, Belgium), Antwerp University Hospital (Antwerp, Belgium), Adelante Centre of Expertise in Rehabilitation (Hoensbroek, The Netherlands) and Maastricht UMC+ (Maastricht, The Netherlands). HC were recruited at Hasselt University (Hasselt, Belgium). The study was approved by the institutional Medical Ethics Committees. Written informed consent was obtained from all participants in accordance with the Declaration of Helsinki.

Peripheral blood was collected from traumatic SCI patients (total cohort: *n* = 70), who were not treated with corticosteroids, and HC (total cohort: *n* = 90). Depending on the type of experiment, subgroups of SCI patients and HC were included in the different experimental analyses (Table 1). SCI samples were included at different time points or phases post-SCI according to Rowland et al. [39]: acute phase (0 weeks (w) post-SCI (within 0–4 days)), subacute phase (2w post-SCI), intermediate phase (3w, 6w, 12w and 18w post-SCI) and chronic phase (26w and 52w post-SCI). Injury severity in the acute phase and at follow-up (most recent available measurement) was measured by the American Spinal Injury Association (ASIA) impairment scale (AIS). The AIS is a standardized neurological examination consisting of a myotomal-based motor examination, dermatomal-based sensory examination and an anorectal examination aimed at determining SCI severity, in which score A represents a complete injury, B a sensory incomplete injury, C a motor incomplete injury with less than half of key muscles below the neurological level having a muscle grade greater than or equal to 3, D a motor incomplete injury with at least half of key muscles below the neurological level having a muscle grade greater than or equal to 3 and E represents a return to normal functioning [40]. HC did not have allergies, acute or chronic injuries, autoimmune disorders, (history of) malignancies or infections at the moment of sampling and were matched to SCI patients with regard to age and sex as closely as possible.

**Table 1:** Study cohort

	MIF ELISA		Absolute immune cell numbers		MIF/CD74 axis screening		*In vitro* blocking assay	
	HC	SCI	HC	SCI	HC	SCI	HC	SCI
N	38	48 (131 samples)	23	27 (90 samples)	47	51 (95 samples)	10	10
Sex (n male, %)	28 (74%)	37 (77%)	18 (78%)	22 (82%)	37 (78%)	40 (78%)	7 (70%)	7 (70%)
Age (years, SD)	59 (16)	56 (17)	60 (17)	62 (15)	56 (17)	57 (17)	48 (17)	47.6 (17)
Level of injury (n, % of patients)								
*Cervical*	/	36 (75%)	/	20 (74%)	/	36 (71%)	/	4 (40%)
* Thoracic*	/	10 (21%)	/	7 (26%)	/	14 (27%)	/	6 (60%)
* Lumbar*	/	1 (2%)	/	0 (0%)	/	1 (2%)	/	0 (0%)
* NA*	/	1 (2%)	/	0 (0%)	/	0 (0%)	/	0 (0%)
AIS score at baseline (0 weeks post-SCI) (n, % of patients)								
* A*	/	12 (25%)	/	10 (37%)	/	17 (33%)	/	8 (80%)
* B*	/	7 (15%)	/	3 (11%)	/	7 (14%)	/	1 (10%)
* C*	/	9 (19%)	/	1 (4%)	/	6 (12%)	/	0 (0%)
* D*	/	18 (38%)	/	13 (48%)	/	19 (37%)	/	1 (10%)
* NA*	/	2 (4%)	/	0 (0%)	/	2 (4%)	/	0 (0%)
AIS score at follow-up (n, % of patients)								
* A*	/	8 (17%)	/	8 (30%)	/	13 (25%)	/	7 (70%)
* B*	/	6 (13%)	/	0 (0%)	/	5 (10%)	/	1 (10%)
* C*	/	4 (8%)	/	3 (11%)	/	5 (10%)	/	1 (10%)
* D*	/	19 (40%)	/	10 (37%)	/	18 (35%)	/	0 (0%)
* E*	/	4 (8%)	/	3 (11%)	/	5 (10%)	/	1 (10%)
* NA*	/	7 (15%)	/	3 (11%)	/	5 (10%)	/	0 (0%)
Included time points post-SCI (n, % of total samples)								
* 0 weeks*	/	29 (22%)	/	16 (18%)	/	31 (33%)	/	0 (0%)
* 2 weeks*	/	12 (9%)	/	10 (11%)	/	5 (5%)	/	0 (0%)
* 3 weeks*	/	22 (17%)	/	10 (11%)	/	22 (23%)	/	5 (50%)
* 6 weeks*	/	25 (19%)	/	11 (12%)	/	10 (11%)	/	5 (50%)
* 12 weeks*	/	21 (16%)	/	9 (10%)	/	0 (0%)	/	0 (0%)
* 18 weeks*	/	11 (8%)	/	8 (9%)	/	0 (0%)	/	0 (0%)
* 26 weeks*	/	11 (8%)	/	14 (16%)	/	15 (16%)	/	0 (0%)
* 52 weeks*	/	0 (0%)	/	12 (13%)	/	12 (13%)	/	0 (0%)

*AIS,* *American Spinal Injury Association (ASIA) impairment scale*. *HC,* *healthy controls*. *MIF,* *macrophage migration inhibitory factor*. *NA,* *not available*. *SCI,* *spinal cord injury*.

### Processing of peripheral blood samples

Whole blood of a subset of donors (Table 1) was used to define absolute numbers of immune cells. Afterwards, peripheral blood mononuclear cells (PBMCs) were isolated from whole blood using Ficoll density gradient centrifugation (Lympholyte^®^ solution, Cedarlane Laboratories, SanBio B.V., Uden, The Netherlands). Plasma was stored at −80°C and PBMCs were cryopreserved in liquid nitrogen at the University Biobank Limburg.

### Human MIF ELISA

MIF plasma levels were measured using the Human MIF DuoSet ELISA Kit (R&D Systems, Bio-Techne, Dublin, Ireland) in half-area 96-well microplates. Before analysis, plasma was diluted in 1x Reagent Diluent Concentrate 2 (R&D Systems, Bio-Techne). All samples were analyzed in duplicate or triplicate. To control for inter-plate variability, the same plasma samples from one HC and one SCI patient were included in duplicate on each ELISA plate as inter-assay controls. The coefficient of variation (CV) of these control samples across plates was below 20%.

### Flow cytometry

#### Absolute numbers of immune cells

Whole blood was incubated with Human TruStain FcX™ (1/20; BioLegend, Amsterdam, The Netherlands) for 10 min at room temperature (RT) and subsequently stained with anti-human antibodies against CD3, CD14, CD19, CD45, CD56 and HLA-DR (Table S1) for 15 min at RT. Next, red blood cells (RBCs) were lysed using 1x RBC Lysis/Fixation solution (BioLegend) for 15 min at RT. Precision Count Beads^™^ (BioLegend) were added according to the manufacturer’s instructions. Samples were acquired using the LSRFortessa flow cytometer (BD Biosciences, Erembodegem, Belgium) and analyzed using FlowJo 10.10.0 (BD Biosciences).

Absolute cell numbers were calculated using the following formula:

$$\:Absolute\:cell\:number\:\left(\raisebox{1ex}{$cells$}\!\left/\:\!\raisebox{-1ex}{$\mu\:l$}\right.\right)=\:\frac{cell\:count}{Precision\:Count\:Beads\:count}\:\times\:\:Precision\:Count\:Beads\:concentration\:\left(\raisebox{1ex}{$beads$}\!\left/\:\!\raisebox{-1ex}{$\mu\:l$}\right.\right)$$

Absolute cell numbers of MIF/CD74 axis-expressing immune cells were calculated as follows (percentage of MIF/CD74 axis-expressing immune cell subsets defined in section “*MIF/CD74 axis screening*”):

$$\:Absolute\:number\:of\:immune\:cell\:subset\:\left(\raisebox{1ex}{$cells$}\!\left/\:\!\raisebox{-1ex}{$\mu\:l$}\right.\right)\times\:Percentage\:of\:MIF\mathrm{/}CD74\:axis\mathrm{-}expressing\:immune\:cell\:subset$$

#### MIF/CD74 axis screening

A 24-color spectral flow cytometry panel was designed and optimized to study the expression of members of the MIF/CD74 axis on 24 distinct immune cell subsets. For this, thawed PBMCs (≤ 2 × 10^6^ cells) were stained with a viability dye and a first set of surface antibodies (Table S2) in 1x phosphate-buffered saline (PBS) for 15 min at RT. Next, the cells were incubated with True-Stain Monocyte Blocker™ and Human TruStain FcX™ (both 1/20; BioLegend) in 1xPBS with 5% fetal bovine serum (FBS; Gibco, ThermoFisher, Waltham, MA, USA) and 0.1% Na-azide (VWR Chemicals, Avantor, Leuven, Belgium) for 10 min at RT. Cells were then incubated with the second set of surface antibodies (Table S3) in 1xPBS with 5% FBS for 15 min at 37°C-5% CO_2_. PBMCs were fixed using BD Cytofix/Cytoperm™ (BD Biosciences) for 20 min at 4°C and incubated overnight at 4°C with anti-human AF647 MIF antibody (1/200; R&D systems, Bio-Techne) in 1x BD Perm/Wash buffer (BD Biosciences).

Samples were acquired using the Cytek^®^ Aurora flow cytometer (Cytek^®^ Biosciences, Amsterdam, The Netherlands) and analyzed using FlowJo 10.10.0 software. The representative gating strategy is shown in Figure S1. Additionally, a multidimensional flow cytometry data analysis was performed. First, single live cells or total CD19^+^ B cells from each individual were randomly down-sampled to 10,000 events, if possible, for general immune cell subsets or B cell subsets, respectively. Individual files were then concatenated into a single file that still allowed the discrimination of the study groups. Dimensionality reduction was performed using the t-Distributed Stochastic Neighbor Embedding (tSNE) native platform in FlowJo with 1,000 iterations.

### *In vitro* blocking of the MIF/CD74 axis

#### Proliferation and activation assay

Human primary B cells were isolated from thawed PBMCs using the EasySep™ Human B Cell Enrichment Kit (STEMCELL Technologies SARL, Saint-Egrève, France), following the manufacturer’s instructions. Purity of the isolated B cells was confirmed on a LSRFortessa flow cytometer following staining with an anti-human BV650 CD19 antibody (1/50; BioLegend) and was routinely > 98%.

Following isolation, primary B cells were labeled with the proliferation dye carboxyfluorescein succinimidyl ester (CFSE; 0.5 µM, BioLegend) for 20 min at 37°C and seeded in 96-well U-bottom plates at 0.5 × 10^5^ cells/condition in culture medium (CM; RPMI-1640, 10% FBS, 1% non-essential amino acids, 1% sodium pyruvate, 50 U/ml penicillin and 50 µg/ml streptomycin (all from Sigma-Aldrich, Merck, Hoeilaart, Belgium)). B cells were stimulated with goat F(ab’)_2_ anti-human IgG/IgA/IgM (1 µg/ml; Jackson ImmunoResearch Europe, Suffolk, UK) and CpG2006 oligonucleotides (ODN 2006, 1 µg/ml; Invivogen, Toulouse, France) for 72 h at 37°C-5% CO_2_. After 24 h, complete stimulation was achieved by adding CD40 ligand (CD40L, 1 µg/ml; BioLegend). Members of the MIF/CD74 axis were blocked separately for 72 h using monoclonal antibodies (CD74, CD44) or a small molecule inhibitor (MIF) (Table S4). After 72 h, B cells were transferred to 96-well V-bottom plates for flow cytometry. Following centrifugation, culture supernatants were stored at −20°C and B cells were stained with Fixable Viability Dye (FVD) eFluor 780 (1/1000; ThermoFisher) in 1xPBS for 30 min at 4°C. Lastly, B cells were stained with anti-human antibodies against CD19, CD80 and CD86 (Table S5) in 1xPBS with 5% FBS and 0.1% Na-azide for 15 min at RT. Flow cytometry was performed on a LSRFortessa flow cytometer and analyzed using FlowJo 10.10.0 software.

#### Cytokine analysis

Levels of IL-1β, TNF-α, IL-6 and IL-10 in the culture supernatant of the *in vitro* proliferation assay was measured using the LEGENDplex™ Human Essential Immune Response Mix and Match Panel (BioLegend), according to the manufacturer’s instructions. Samples were acquired with the LSRFortessa flow cytometer and analyzed using the online LEGENDplex™ software tool (BioLegend).

### Statistical analysis

Data analysis of absolute cell numbers, MIF/CD74 axis screening and human MIF ELISA measurements was performed using JMP Pro 17.2.0 software (SAS Institute, Cary, NC, USA). Continuous variables were log10-transformed, if necessary, to improve distributional symmetry and approximate a Gaussian distribution. In SCI patients, changes over time in absolute cell numbers, MIF/CD74 axis expression and MIF levels were analyzed using linear mixed-effects models, with time included as a categorical fixed effect. When a significant effect of time was observed, post-hoc analyses were performed using Tukey HSD tests for pairwise multiple comparisons. Linear mixed-effects models were also used to assess overall differences in MIF levels between HC and SCI patients (across all time points), with group (HC vs. SCI) included as a fixed effect. All mixed-effects models accounted for repeated measurements by including patient ID as a random effect. Additional fixed effects included age, sex, injury severity at baseline (AIS at 0w post-SCI) and injury level (cervical, thoracic or lumbar). Backward stepwise selection was applied, with effects showing *p* > 0.05 sequentially removed, while retaining the primary variable of interest (time or group) in the model. Absolute numbers, MIF/CD74 axis expression and MIF levels of SCI patients at each time point were compared with HC, serving as the reference group, using a Steel test for nonparametric multiple comparisons.

Data analysis of the *in vitro* blocking assay was done using Prism software version 10.4.2 (GraphPad, San Diego, CA, USA). Normality was checked using the Shapiro-Wilk test. Differences in proliferation and expression of CD80, CD86, IL-1β, IL-6, IL-10 and TNF-α between the blocked and control conditions were analyzed using one-tailed t-tests or Wilcoxon signed-rank tests. Differences in proliferation, CD80, CD86, IL-1β, IL-6, IL-10 and TNF-α expression between HC and SCI patients were analyzed using one-tailed t-tests or Mann-Whitney tests. A p-value of < 0.05 was considered significant. Prism software version 10.4.2 was used for graphical representation.

## Results

### MIF levels are increased in the plasma of traumatic SCI patients

**Fig. 1:**
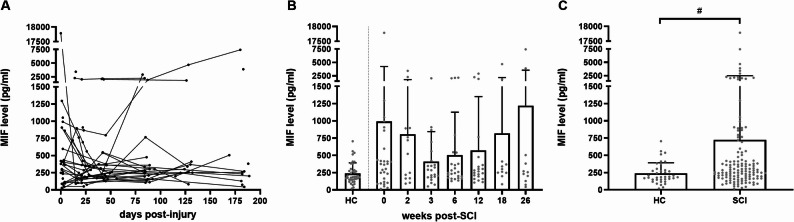
MIF plasma levels in HC and SCI patients. **A** MIF plasma levels in SCI patients (*n* = 48) over time (days post-injury). Values from the same patient are connected with a line. **B-C** MIF plasma levels in HC (*n* = 38) and SCI patients (*n* = 48) at 0 (0-4dpi; *n* = 29), 2 (12-20dpi; *n* = 12), 3 (21-33dpi; *n* = 22), 6 (36-56dpi; *n* = 25), 12 (82-97dpi; *n* = 21), 18 (115-132dpi; *n* = 11) and 26 (169-189dpi; *n* = 11) weeks post-SCI. Mean (± SD) is depicted. Differences in SCI patients over time (**A**) and between HC and SCI patients across all time points (**C**, depicted with a hash mark) were analyzed using linear mixed-effects models. SCI measurements at each time point (**B**) were compared with HC (reference group) using a Steel test for nonparametric multiple comparisons. ^*#*^*p* < 0.05. *Dpi, days post-injury. HC*,* healthy controls. MIF*,* macrophage migration inhibitory factor. NK cells*,* natural killer cells. SCI*,* spinal cord injury.*

In a previous study, we reported a potential role for the MIF/CD74 axis in post-SCI B cell responses [38]. However, MIF plasma levels were similar in HC and SCI patients, possibly because of the smaller patient cohort that was available at that time [38]. Therefore, we now measured MIF plasma levels in a larger longitudinal SCI patient cohort (*n* = 48), as well as in HC (*n* = 38) (Table 1). Although we did not detect a significant change in MIF plasma levels over time in SCI patients (Fig. 1A-B), MIF levels were significantly increased in SCI patients compared to HC (*p* = 0.0122) (Fig. 1C). While no significant differences were observed over time, the increased MIF plasma levels in SCI patients indicate that MIF could regulate B cell responses throughout the different phases post-SCI.

### Early immune cell reduction post-SCI affects absolute numbers of MIF/CD74 axis-expressing immune cells

Next, we aimed to map the entire MIF/CD74 axis across the peripheral immune system. Therefore, we first analyzed the absolute numbers of major immune cell subsets expressing members of the MIF/CD74 axis. Within CD45^+^ cells, we analyzed numbers of CD19^+^ B cells, CD14^+^ monocytes, HLA-DR^+^ DCs, CD56^+^ natural killer (NK) cells and CD3^+^ T cells of 23 HC and 27 SCI patients (0w: *n* = 16, 2w: *n* = 10, 3w: *n* = 10, 6w: *n* = 11, 12w: *n* = 9, 18w *n* = 8, 26w: *n* = 14, 52w: *n* = 12) (Table 1). Additionally, we determined the absolute numbers of B cells, monocytes, DCs, NK cells and T cells expressing MIF, as well as B cells expressing MIF receptors, in HC (*n* = 13) and SCI patients (*n* = 20) at the acute (*n* = 13), subacute/intermediate (*n* = 11) and chronic (*n* = 13) phases post-injury.

**Fig. 2:**
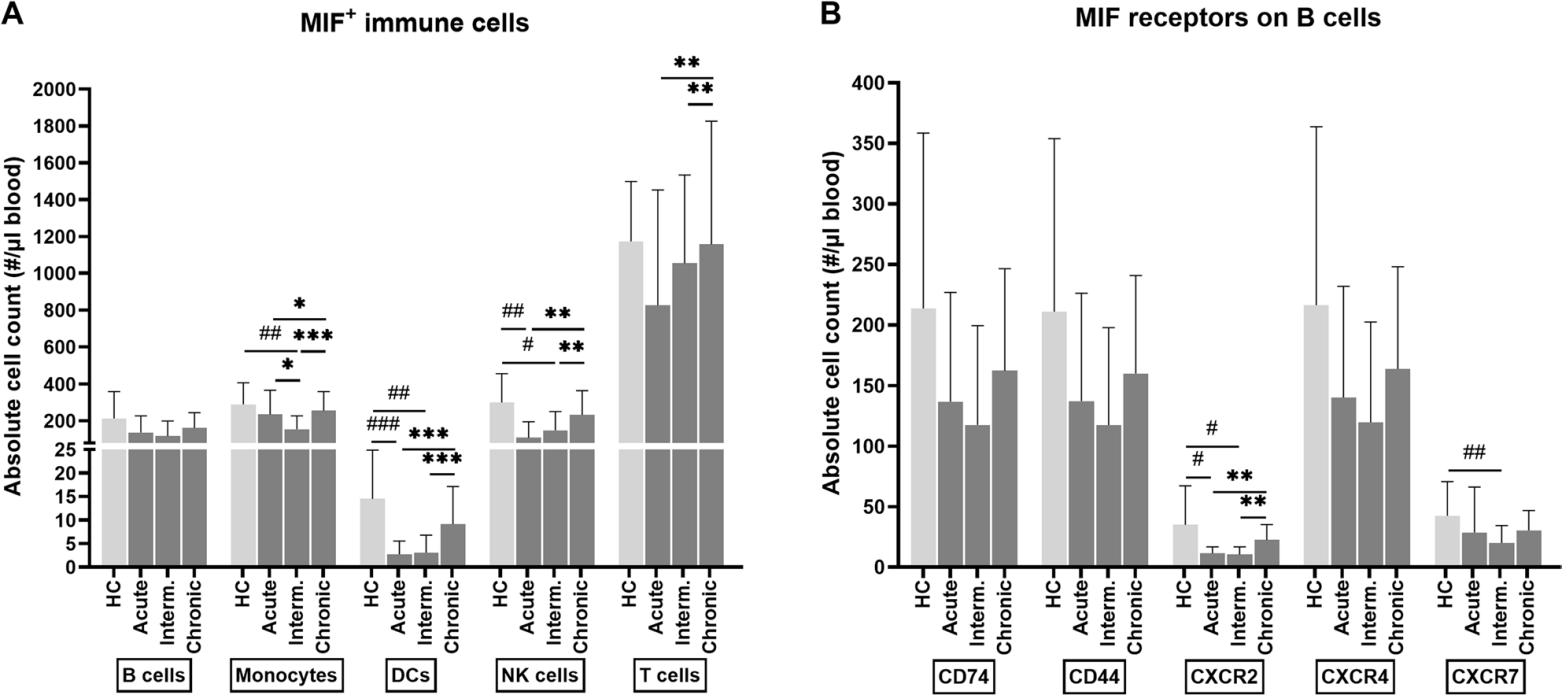
Absolute numbers of MIF/CD74 axis-expressing immune cells in HC and SCI patients. Absolute numbers of (**A**) B cells, monocytes, DCs, NK cells and T cells expressing MIF, and (**B**) B cells expressing CD74, CD44, CXCR2, CXCR4 and CXCR7 in HC (*n* = 13) and SCI patients (*n* = 20) at the acute (1-4dpi; *n* = 13), subacute/intermediate (18-46dpi; *n* = 11) and chronic (178-379dpi; *n* = 13) phases post-SCI. Mean (± SD) is depicted. Differences in SCI patients over time were analyzed using linear mixed-effects models and post-hoc Tukey HSD tests, and depicted in the figure using asterisks. SCI measurements at each time point were compared with HC (reference group) using a Steel test for nonparametric multiple comparisons, and depicted in the figure using hash marks. *^/#^*p* < 0.05, **^/##^*p* < 0.01, ***^/###^*p* < 0.001. *CXCR, CXC-motif chemokine receptor. DCs*,* dendritic cells. Dpi*,* days post-injury. HC*,* healthy controls. Interm.*,* subacute/intermediate. MIF*,* macrophage migration inhibitory factor. NK cells*,* natural killer cells. SCI*,* spinal cord injury.*

In general, absolute immune cell numbers decreased in the acute and subacute/intermediate phases post-SCI when compared to HC and then gradually increased in the chronic phase (Fig. 2, Fig. S2). For MIF-expressing immune cells, absolute numbers of MIF^+^ monocytes, DCs and NK cells were significantly reduced in the acute (DCs: *p* = 0.0004, NK cells: *p* = 0.0010) and subacute/intermediate (monocytes: *p* = 0.0061, DCs: *p* = 0.0015, NK cells: *p* = 0.0151) phases post-injury compared to HC (Fig. 2A). MIF^+^ cell numbers increased over time and were significantly higher in the chronic phase compared to the acute and subacute/intermediate phases post-injury for monocytes (*p* = 0.0302 and *p* = 0.0008, respectively), DCs (*p* = 0.0003 and *p* = 0.0002, respectively), NK cells (*p* = 0.0011 and *p* = 0.0065, respectively) and T cells (*p* = 0.0032 and *p* = 0.0094, respectively) (Fig. 2A). No significant changes were observed for MIF^+^ B cells, although they followed the same trend over time. Additionally, the main immune cell subsets followed the same pattern over time, with lower absolute cell numbers at the early time points in SCI patients compared with HC (Fig. S2B-E). Over time, immune cell numbers gradually recovered to some extent, although DC cell numbers remained significantly lower in SCI patients compared to HC over the follow-up period (Fig. S2).

For MIF receptor-expressing B cells, absolute numbers of CXCR2^+^ and CXCR7^+^ B cells were significantly lower in SCI patients in the acute (CXCR2: *p* = 0.0283) and subacute/intermediate (CXCR2: *p* = 0.0151, CXCR7: *p* = 0.0251) phases post-SCI compared to HC (Fig. 2B). Numbers of CXCR2^+^ and CXCR7^+^ B cells increased again in the chronic phase post-SCI, but this was only significant for CXCR2^+^ B cells (vs. acute: *p* = 0.0057, vs. subacute/intermediate: *p* = 0.0031). The timing of these changes corresponded with the time points at which total B cell numbers were decreased in SCI patients compared to HC (2w: *p* = 0.0372, 3w: *p* = 0.0468) (Fig. S2A). No significant changes were observed for CD74^+^, CD44^+^, CXCR4^+^ and MIF^+^ B cells, although they followed the same trend over time (Fig. 2B).

Our data shows that the absolute numbers of MIF^+^ immune cells, as well as B cells expressing members of the MIF/CD74 axis, were decreased up to the intermediate phase post-SCI, but gradually increased over time when proceeding to the chronic phase. This may point to a rapid immune cell reduction post-SCI that affects the MIF/CD74 axis-expressing immune cells.

### The immune landscape post-SCI shows early B cell, T cell and monocyte increases, selective loss of transitional and unswitched memory B cells, and antibody-secreting cell upregulation

**Fig. 3:**
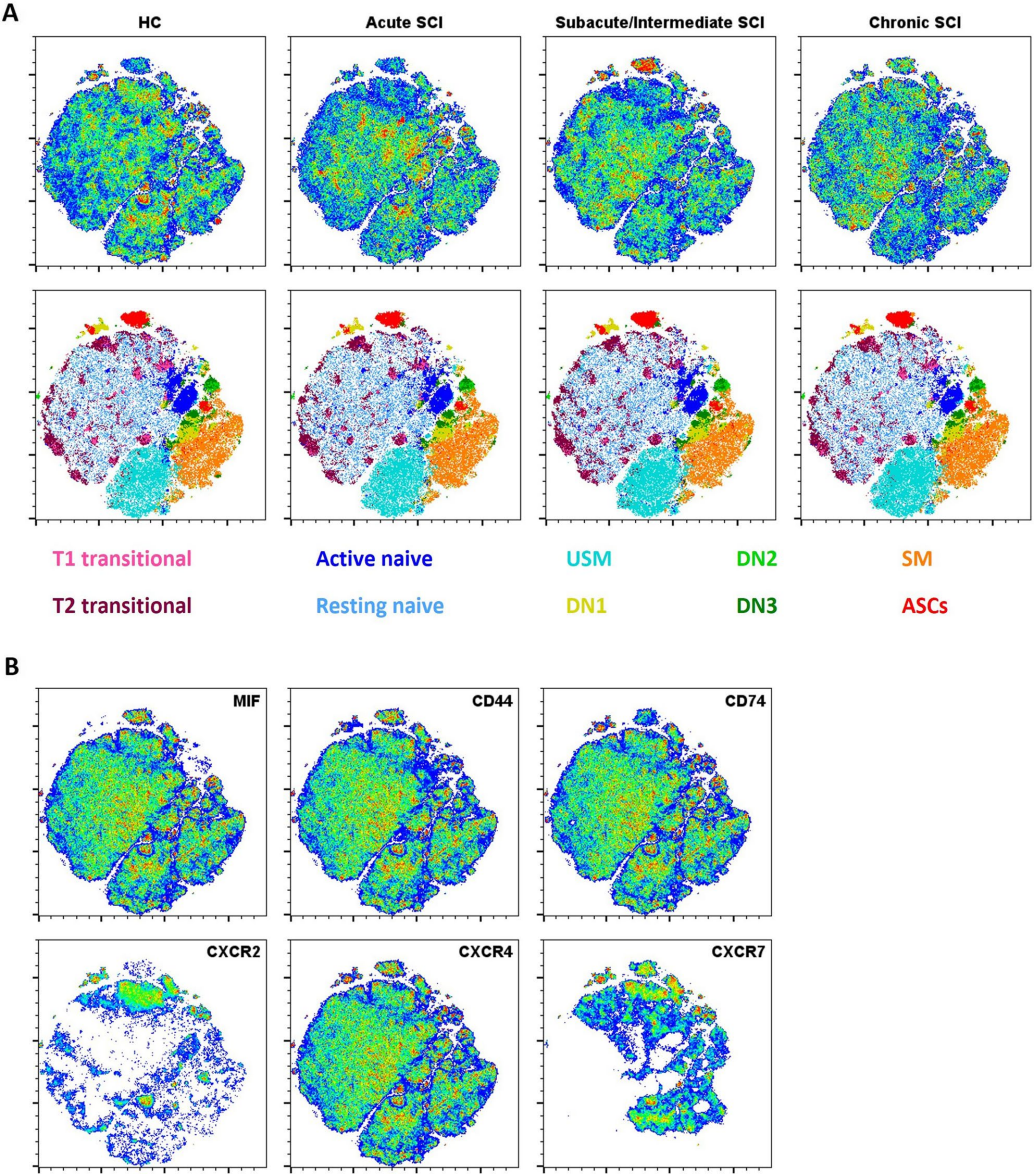
High-dimensional flow cytometry of B cells and the MIF/CD74 axis in HC and SCI patients. **A** tSNE maps showing the B cell subsets within CD19^+^ B cells of HC (*n* = 47) and SCI patients (*n* = 51) at the acute (0-4dpi; *n* = 31), subacute/intermediate (16-46dpi; *n* = 37) and chronic (189-379dpi; *n* = 27) phases post-SCI. The upper row shows the tSNE maps on pseudocolor plots, while the lower row shows the manually determined gates on the tSNE maps. **B** tSNE maps (pseudocolor plots) showing the expression of MIF, CD44, CD74, CXCR2, CXCR4 and CXCR7 within CD19^+^ B cells (*n* = 98). *ASCs*,* antibody-secreting cells. DN*,* double negative. Dpi*,* days post-injury. HC*,* healthy control. SCI*,* spinal cord injury. SM*,* switched memory. tSNE*,* t-Distributed Stochastic Neighbor Embedding. USM*,* unswitched memory.*

To study the proportional levels of MIF-expressing immune cell subsets and MIF receptor-expressing B cell subsets, high-dimensional spectral flow cytometry was used. Analysis was focused on MIF and its receptors CD74, CD44, CXCR2, CXCR4 and CXCR7 within detailed immune cell and B cell subsets. An overview of all included immune cell and B cell subsets, with their corresponding surface marker expression, is provided in Figure S1. PBMC of HC (*n* = 47) and SCI patients (*n* = 51) at the acute (*n* = 31), subacute/intermediate (*n* = 37) and chronic (*n* = 27) phases post-SCI were included (Table 1). A tSNE algorithm was used in an exploratory manner to visualize the major immune cell subsets (Fig. S3) or B cell subsets (Fig. 3) and the MIF/CD74 axis. Shifts in the cell densities were evident on the pseudocolor tSNE plots, indicating changes in both general immune cell (Fig. S3A) and B cell subset (Fig. 3A) distribution between the study groups. An overlay of the manually gated cells on the tSNE maps displayed separate clustering of the major immune cell and B cell subsets. For B cell analysis, transitional and naive B cells clustered closely together, as well as switched memory (SM), unswitched memory (USM) and double negative (DN)1 cells (Fig. 3A). DN2 cells and antibody-secreting cells (ASCs) formed separate clusters, while DN3 cells were interspersed between DN1, DN2 and SM B cells.

**Fig. 4:**
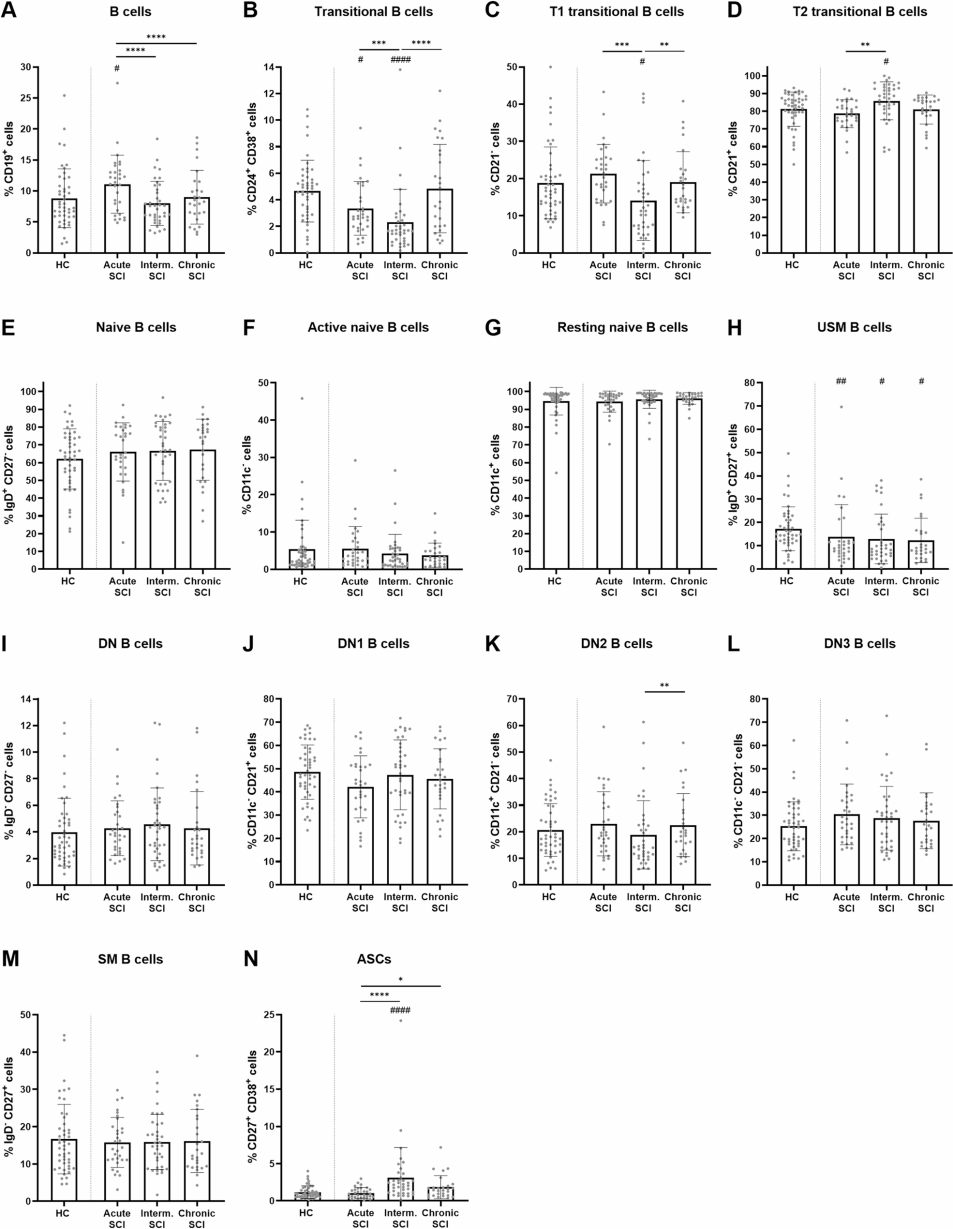
Percentage of B cell subsets in HC and SCI patients, at different phases post-SCI. Percentage of total B cells (**A**), transitional B cells (**B**), including T1 (**C**) and T2 (**D**) transitional B cells, naive B cells (**E**), including active (**F**) and resting (**G**) naive B cells, USM B cells (**H**), DN B cells (**I**), including DN1 (**J**), DN2 (**K**) and DN3 (**L**) B cells, SM B cells (**M**) and ASCs (**N**) in HC (*n* = 47) and SCI patients (*n* = 51) at the acute (0-4dpi; *n* = 31), subacute/intermediate (16-46dpi; *n* = 37) and chronic (189-379dpi; *n* = 27) phases post-SCI. Mean (± SD) is depicted. Differences in SCI patients over time were analyzed using linear mixed-effects models and post-hoc Tukey HSD tests, and depicted in the figure using asterisks. SCI measurements at each time point were compared with HC (reference group) using a Steel test for nonparametric multiple comparisons, and depicted in the figure using hash marks. *^/#^*p* < 0.05, **^/##^*p* < 0.01, ***p < 0.001, ****^/####^*p* < 0.0001. *ASCs, antibody-secreting cells. DN B cells*,* double negative B cells. Dpi*,* days post-injury. HC*,* healthy controls. Interm.*,* subacute/intermediate. SCI*,* spinal cord injury. SM B cells*,* switched memory B cells. USM B cells*,* unswitched memory B cells.*

As the tSNE plots showed shifts in cell densities, we first compared the frequencies of the immune cell and B cell subsets between the study groups. Total CD19^+^ B cell frequencies were elevated in the acute phase post-SCI, both when compared to the subacute/intermediate and chronic phases (both *p* < 0.0001), and to HC (*p* = 0.0475) (Fig. 4A). For the other immune cell subsets, total CD3^+^ T cells (acute and subacute/intermediate phases), CD14^hi^CD16^-^ classical monocytes (acute and chronic phases) and CD14^hi^CD16^hi^ intermediate monocytes (acute phase) also showed increased frequencies in SCI patients compared to HC (Fig. S4). Interestingly, the frequency of intermediate monocytes decreased again in the subacute/intermediate and chronic phases post-SCI. In contrast, frequencies of CD14^lo^CD16^hi^ non-classical monocytes (all time points), CD56^-^ DCs (acute and subacute/intermediate phases) and CD56^+^ NK cells (acute and subacute/intermediate phases) were decreased in SCI patients compared to HC (Fig. S4).

When looking into the different B cell subsets, total transitional B cells were significantly reduced in the acute (*p* = 0.0253) and subacute/intermediate (*p* < 0.0001) phases post-SCI when compared to HC, and in the subacute/intermediate phase compared to the acute (*p* = 0.0007) and chronic (*p* < 0.0001) phases post-SCI (Fig. 4B). This decrease in transitional B cells in the subacute/intermediate phase was due to a downregulation of T1 B cells (vs. acute: *p* = 0.0002, vs. chronic: *p* = 0.0018, vs. HC: *p* = 0.0232), whereas T2 B cells were upregulated (vs. acute: *p* = 0.0041, vs. HC: *p* = 0.0235) (Fig. 4C, D). In all phases post-SCI, the frequency of USM B cells was decreased compared to HC (acute: *p* = 0.0087, subacute/intermediate: *p* = 0.0134, chronic: *p* = 0.0204) (Fig. 4H). Within DN B cells, the percentage of DN2 cells were decreased in the subacute/intermediate versus the chronic phase post-SCI (*p* = 0.0035) (Fig. 4K). Lastly, ASC frequencies were significantly increased in the subacute/intermediate and chronic phases post-SCI compared to the acute phase (*p* < 0.0001 and *p* = 0.0240, respectively) and to HC (vs. subacute/intermediate phase: *p* < 0.0001) (Fig. 4N). No changes were observed for naive, DN and SM B cells (Fig. 4E-G, I, M).

Thus, although all immune cell subsets showed an early decrease in absolute numbers post-SCI, differential shifts in their relative distribution were present, with increased frequencies of B cells, T cells and monocytes, and decreased frequencies of DCs and NK cells in the acute and subacute/intermediate phases post-SCI. Within B cell subsets, transitional, USM and DN2 B cell frequencies were reduced, whereas the frequency of ASCs was increased, mainly during the subacute/intermediate phase post-SCI.

### Intracellular MIF expression is reduced within immune cell subsets of SCI patients

MIF has been shown to be upregulated in the injured spinal cord, primarily in astrocytes and microglia, following traumatic SCI [26]. Our data also showed that MIF plasma levels were increased following traumatic SCI. Therefore, we determined whether this cytokine is also upregulated in the peripheral immune system and aimed to identify the MIF-producing immune cell subsets in the circulation of SCI patients and HC. For this, we analyzed MIF expression in different B cell subsets, as well as in major immune cell populations, using our spectral flow cytometry approach. Interestingly, tSNE plots showed MIF expression on all major immune cell and B cell subsets, while its receptor CD74 was mainly present on antigen-presenting cells, such as B cells, DCs and monocytes (Fig. S3B, Fig. 3B). Within the B cell population, MIF receptors CD74, CD44 and CXCR4 were expressed throughout all subsets (Fig. 3B). The other MIF receptors, namely CXCR2 and CXCR7, were expressed more selectively: CXCR2 on a subpopulation of resting naive B cells, transitional and DN B cells, and CXCR7 on ASCs and subpopulations of all other B cell subsets (Fig. 3B).

**Fig. 5:**
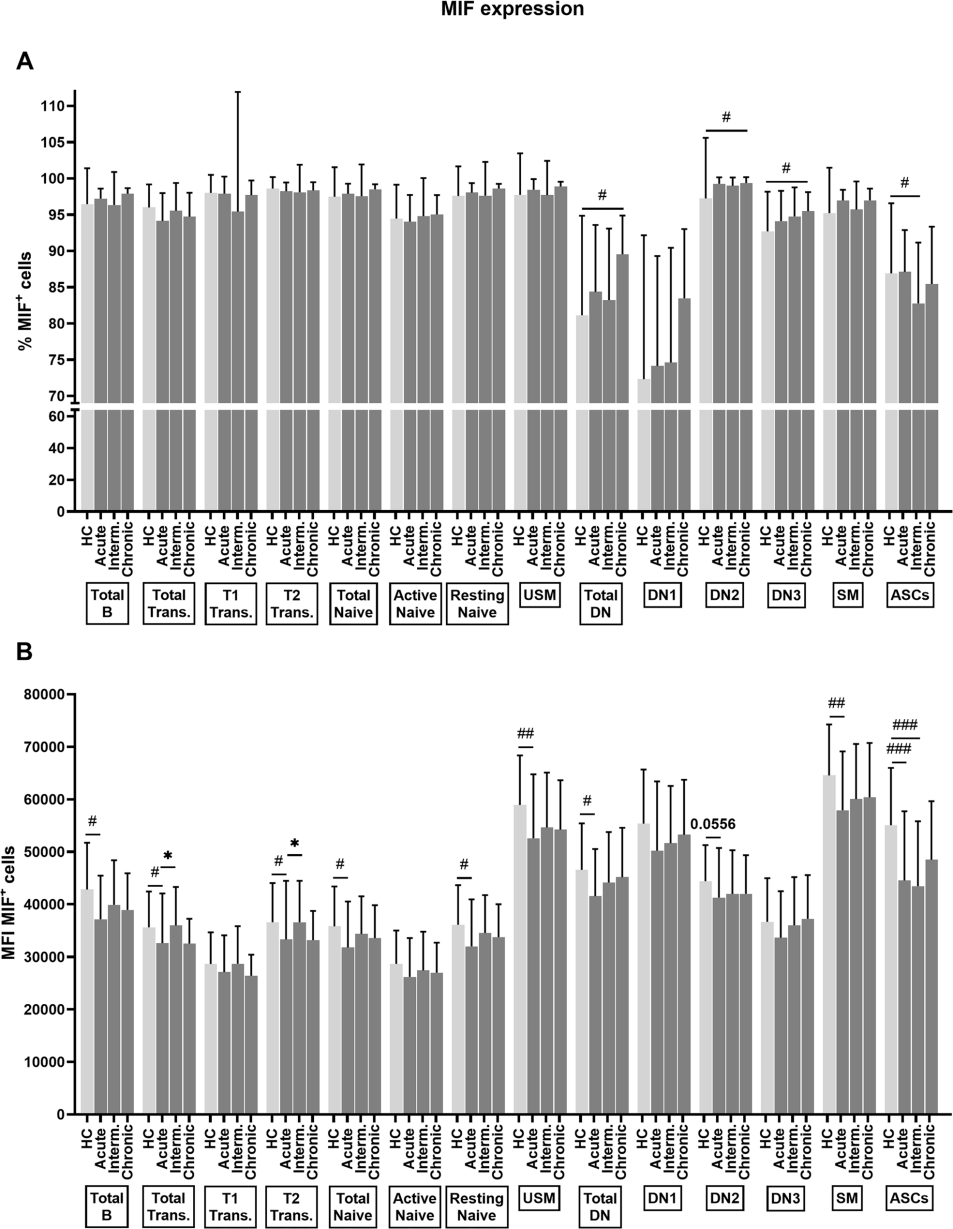
MIF expression in B cell subsets of HC and SCI patients, at different phases post-SCI. Percentage (**A**) and MFI (**B**) of MIF^+^ cells within total B cells, total transitional, T1 transitional, T2 transitional, total naive, active naive, resting naive, USM, total DN, DN1, DN2, DN3, SM B cells and ASCs in HC (*n* = 47) and SCI patients (*n* = 51) at the acute (0-4dpi; *n* = 31), subacute/intermediate (16-46dpi; *n* = 37) and chronic (189-379dpi; *n* = 27) phases post-SCI. Mean (± SD) is depicted. Differences in SCI patients over time were analyzed using linear mixed-effects models and post-hoc Tukey HSD tests, and depicted in the figure using asterisks. SCI measurements at each time point were compared with HC (reference group) using a Steel test for nonparametric multiple comparisons, and depicted in the figure using hash marks. *^/#^*p* < 0.05, ^*##*^*p* < 0.01, ^*###*^*p* < 0.001. *ASCs, antibody-secreting cells. CXCR*,* CXC-motif chemokine receptor. DN*,* double negative. Dpi*,* days post-injury. HC*,* healthy controls. Interm.*,* subacute/intermediate. MFI*,* median fluorescence intensity. SCI*,* spinal cord injury. SM*,* switched memory. Trans.*,* transitional. USM*,* unswitched memory.*

**Fig. 6:**
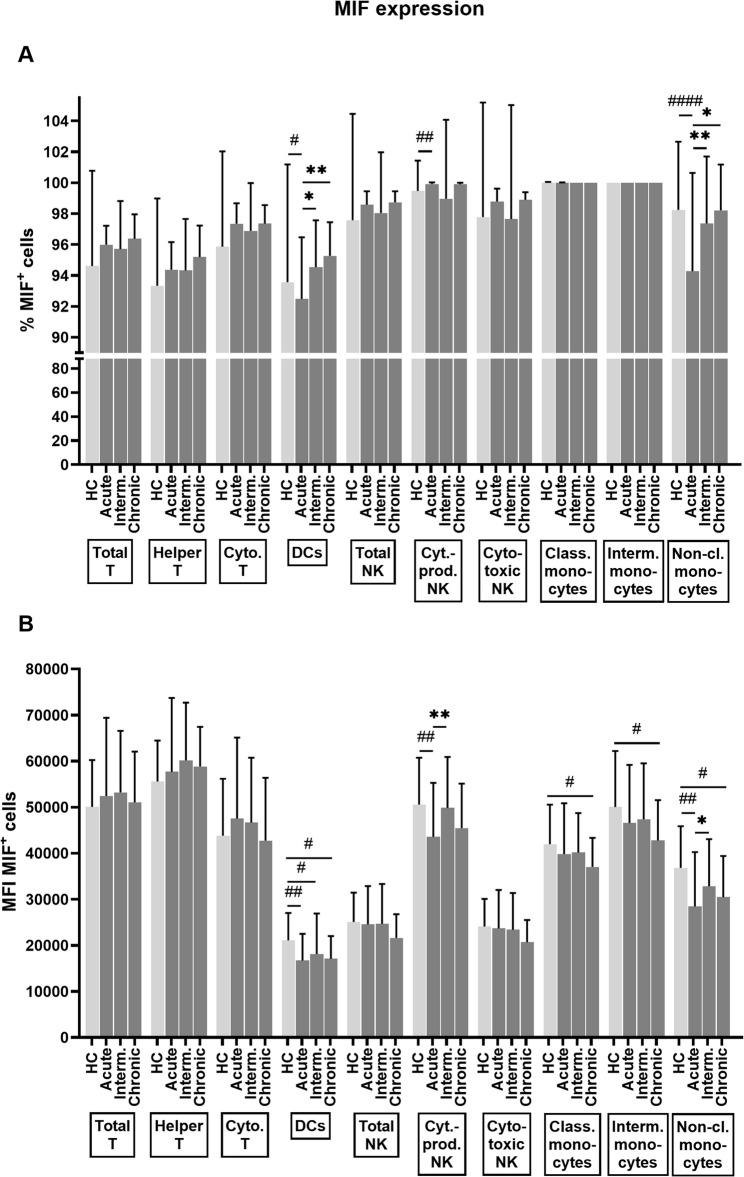
MIF expression in immune cell subsets of HC and SCI patients, at different phases post-SCI. Percentage (**A**) and MFI (**B**) of MIF^+^ cells within total T cells, Th cells, CTLs, DCs, total NK cells, cytokine-producing NK cells, cytotoxic NK cells, classical monocytes, intermediate monocytes and non-classical monocytes in HC (*n* = 47) and SCI patients (*n* = 51) at the acute (0-4dpi; *n* = 31), subacute/intermediate (16-46dpi; *n* = 37) and chronic (189-379dpi; *n* = 27) phases post-SCI. Mean (± SD) is depicted. Differences in SCI patients over time were analyzed using linear mixed-effects models and post-hoc Tukey HSD tests, and depicted in the figure using asterisks. SCI measurements at each time point were compared with HC (reference group) using a Steel test for nonparametric multiple comparisons, and depicted in the figure using hash marks. *^/#^*p* < 0.05, **^/##^*p* < 0.01 ^*####*^*p* < 0.0001. *CTLs, cytotoxic T cells. DCs*,* dendritic cells. Dpi*,* days post-injury. HC*,* healthy control. Interm.*,* subacute/intermediate. MFI*,* median fluorescence intensity. MIF*,* macrophage migration inhibitory factor. NK cells*,* natural killer cells. Th cells*,* helper T cells. SCI*,* spinal cord injury.*

Similar to the tSNE analysis, we observed that the majority of immune cells were MIF^+^ (Figs. 5A and 6A). For the B cell subsets, significant differences between SCI patients and HC were evident for DN B cells and ASCs, whereby MIF^+^ DN B cell subsets were significantly elevated in chronic SCI (total DN: *p* = 0.0139, DN2: *p* = 0.0323, DN3: *p* = 0.0371) and MIF^+^ ASCs were decreased in subacute/intermediate SCI (*p* = 0.0188) (Fig. 5A). The intracellular expression level of MIF (defined as median fluorescence intensity [MFI]) in total B cells and B cell subsets was decreased in SCI patients compared to HC, especially in the acute phase (Fig. 5B, *p*-values in Table S6). In the subacute/intermediate phase, MIF expression in SCI B cells tended to increase again, which was significant for total and T2 transitional B cells, but was still reduced in the subacute/intermediate phase for ASCs (*p*-values in Table S6).

Within the major immune cell populations, the frequency of MIF^+^ cells was significantly increased in acute SCI compared to HC for cytokine-producing NK cells (Fig. 6A, *p*-values in Table S6). In contrast, MIF^+^ DCs and non-classical monocytes were reduced in the acute phase but were increased again in the subacute/intermediate and chronic phases. The other immune cell populations did not show differences in the frequency of MIF^+^ cells (Fig. 6A). Regarding intracellular MIF expression, no significant differences were observed within T cell subsets (Fig. 6B). In DCs, however, MIF expression was significantly decreased at all time points post-SCI compared to HC (Fig. 6B, *p*-values in Table S6). Within cytokine-producing NK cells and non-classical monocytes, intracellular MIF expression was significantly reduced in the acute phase, then increased in the subacute/intermediate phase, but was again reduced in the chronic phase for non-classical monocytes (Fig. 6B, *p*-values in Table S6). For classical and intermediate monocytes, MIF expression was decreased in chronic SCI compared to HC (*p* = 0.0459 and *p* = 0.0377, respectively) (Fig. 6B).

Our data suggest that the proportional levels of MIF^+^ cells were only mildly affected post-SCI. However, intracellular MIF expression levels were reduced in SCI patients compared to HC, suggesting that the peripheral immune system might not be the main source of MIF production post-SCI.

### MIF receptor expression is upregulated on B cells of SCI patients

**Fig. 7:**
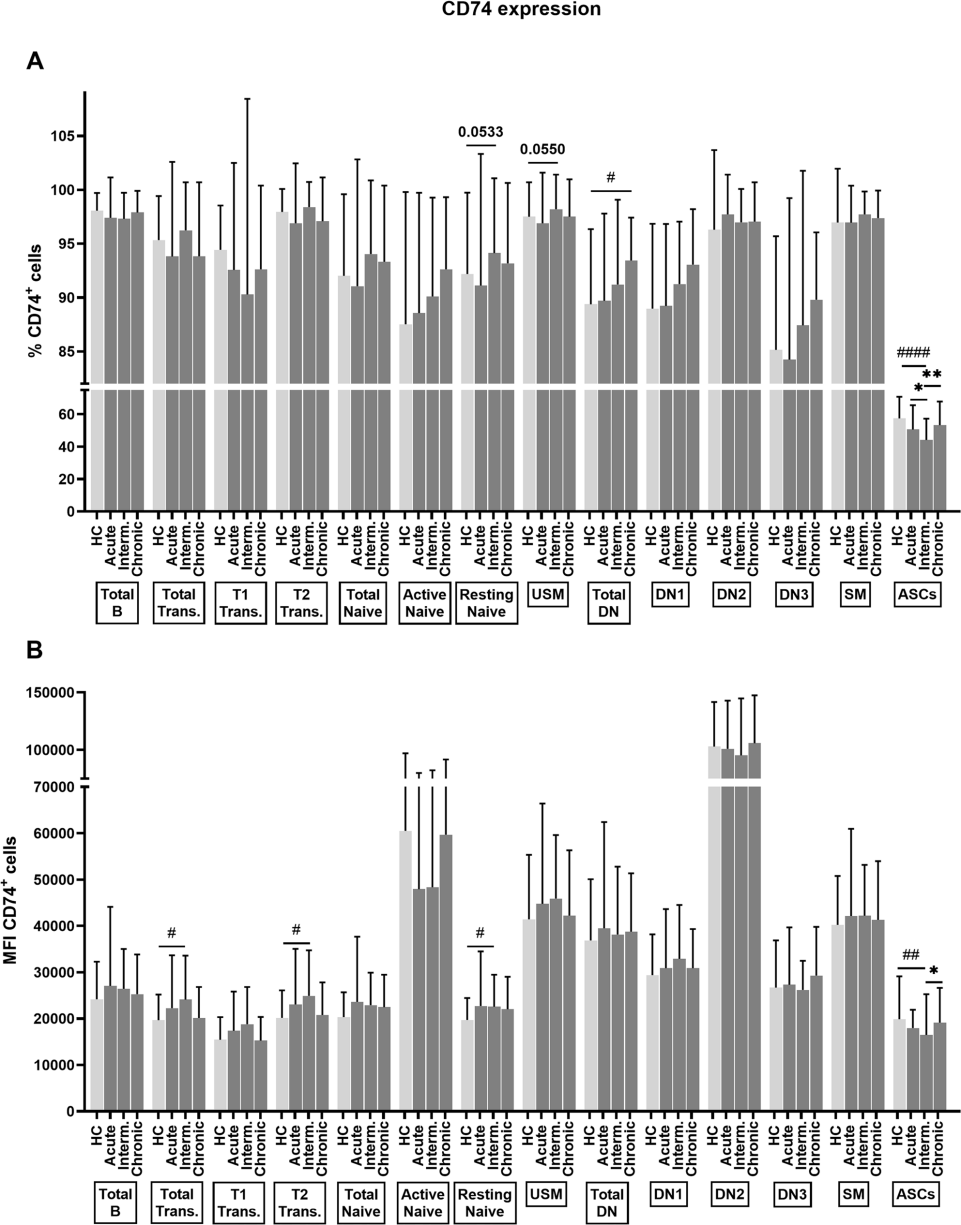
CD74 expression on B cell subsets of HC and SCI patients, at different phases post-SCI. Percentage (**A**) and MFI (**B**) of CD74^+^ cells within total B cells, total transitional, T1 transitional, T2 transitional, total naive, active naive, resting naive, USM, total DN, DN1, DN2, DN3, SM B cells and ASCs in HC (*n* = 47) and SCI patients (*n* = 51) at the acute (0-4dpi; *n* = 31), subacute/intermediate (16-46dpi; *n* = 37) and chronic (189-379dpi; *n* = 27) phases post-SCI. Mean (± SD) is depicted. Differences in SCI patients over time were analyzed using linear mixed-effects models and post-hoc Tukey HSD tests, and depicted in the figure using asterisks. SCI measurements at each time point were compared with HC (reference group) using a Steel test for nonparametric multiple comparisons, and depicted in the figure using hash marks. *^/#^*p* < 0.05, **^/##^*p* < 0.01, ^*####*^*p* < 0.0001. *ASCs, antibody-secreting cells. DN*,* double negative. Dpi*,* days post-injury. HC*,* healthy controls. Interm.*,* subacute/intermediate. MFI*,* median fluorescence intensity. SCI*,* spinal cord injury. SM*,* switched memory. Trans.*,* transitional. USM*,* unswitched memory.*

To provide a comprehensive characterization of the MIF/CD74 axis distribution post-SCI, we subsequently analyzed the frequency of B cell subsets expressing the MIF receptors CD74, CD44, CXCR2, CXCR4 and CXCR7, as well as MIF receptor surface expression (MFI) on B cell subsets. Although the total B cell population did not show differences in the frequency of CD74^+^ cells or CD74 surface expression between the study groups, CD74^+^ cell frequencies and/or CD74 surface expression levels were increased in the subacute/intermediate phase post-SCI when compared to HC within multiple B cell subsets, including total and T2 transitional and resting naive B cells (Fig. 7, *p*-values in Table S7). For total DN B cells, CD74^+^ cell frequencies were increased in the chronic phase post-SCI compared to HC. In contrast, CD74^+^ cells and CD74 expression levels were decreased in ASCs in the subacute/intermediate phase post-SCI compared to HC, and increased again in the chronic phase. Remarkably, active naive and DN2 B cells exhibited higher CD74 expression levels compared to the other B cell subsets.

**Fig. 8:**
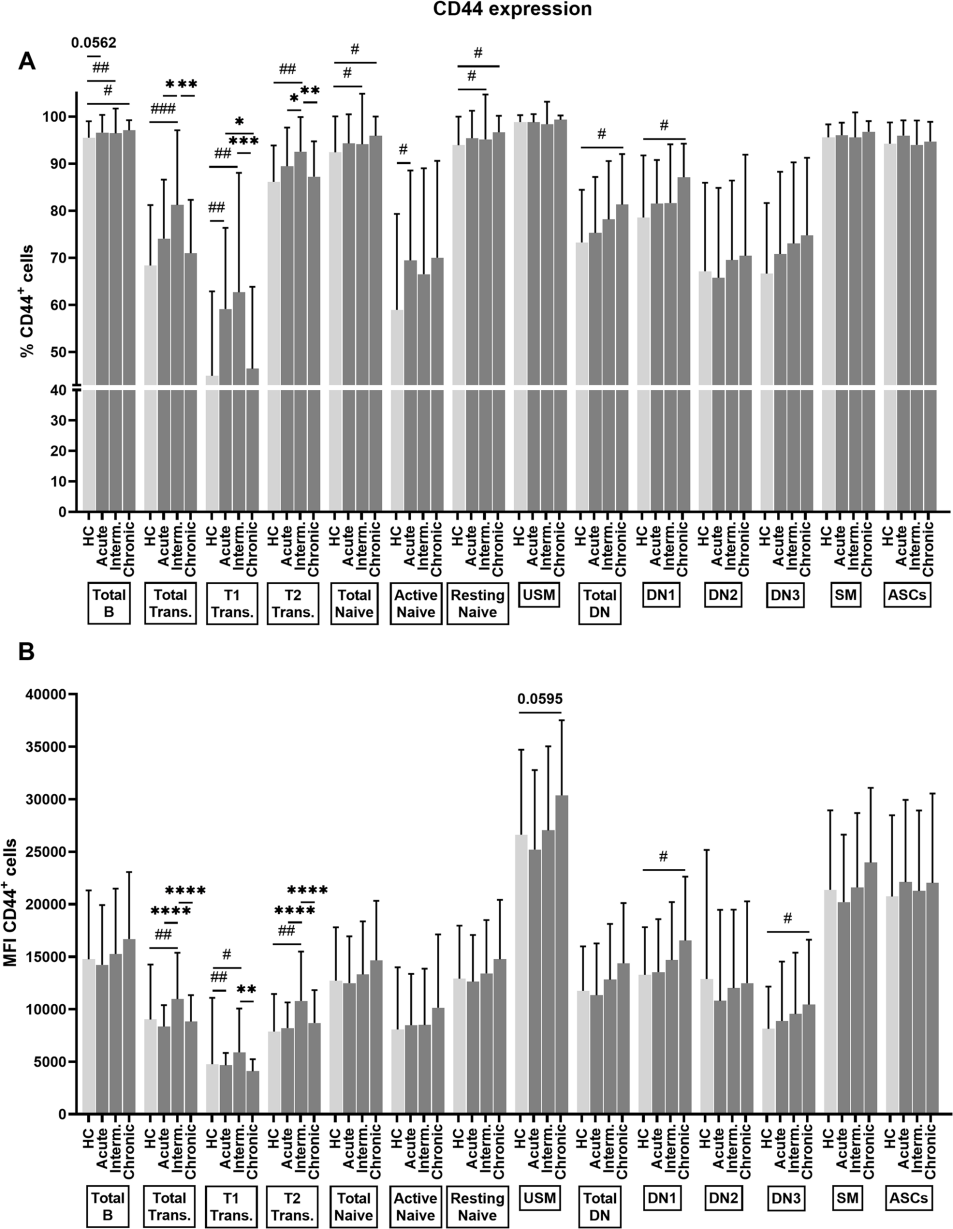
CD44 expression on B cell subsets of HC and SCI patients, at different phases post-SCI. Percentage (**A**) and MFI (**B**) of CD44^+^ cells within total B cells, total transitional, T1 transitional, T2 transitional, total naive, active naive, resting naive, USM, total DN, DN1, DN2, DN3, SM B cells and ASCs in HC (*n* = 47) and SCI patients (*n* = 51) at the acute (0-4dpi; *n* = 31), subacute/intermediate (16-46dpi; *n* = 37) and chronic (189-379dpi; *n* = 27) phases post-SCI. Mean (± SD) is depicted. Differences in SCI patients over time were analyzed using linear mixed-effects models and post-hoc Tukey HSD tests, and depicted in the figure using asterisks. SCI measurements at each time point were compared with HC (reference group) using a Steel test for nonparametric multiple comparisons, and depicted in the figure using hash marks. *^/#^*p* < 0.05, **^/##^*p* < 0.01, ***^/###^*p* < 0.001, ****p < 0.0001. *ASCs, antibody-secreting cells. DN*,* double negative. Dpi*,* days post-injury. HC*,* healthy controls. Interm.*,* subacute/intermediate. MFI*,* median fluorescence intensity. SCI*,* spinal cord injury. SM*,* switched memory. Trans.*,* transitional. USM*,* unswitched memory.*

For the CD74 co-receptor, CD44, overall, most B cell subsets presented with an increased frequency of CD44^+^ cells and CD44 surface expression in SCI patients compared to HC (Fig. 8, *p*-values in Table S8). CD44^+^ cell frequencies and CD44 expression levels tended to increase over time in SCI patients with peak levels in the subacute/intermediate (transitional B cells) or chronic (naive, DN B cells) phases. No differences in the frequency of CD44^+^ cells or CD44 surface expression were present between the study groups for SM B cells or ASCs.

**Fig. 9:**
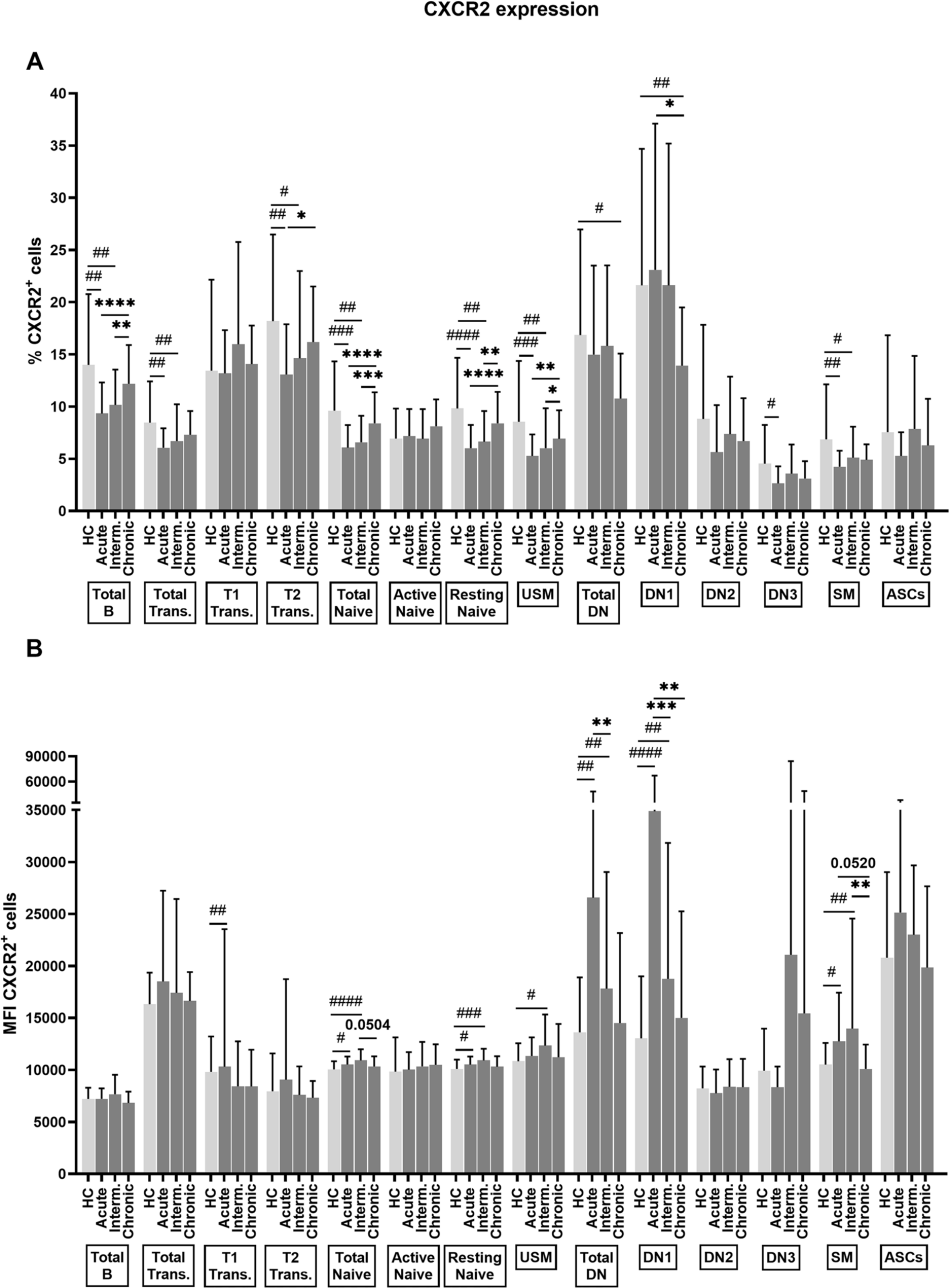
CXCR2 expression on B cell subsets of HC and SCI patients, at different phases post-SCI. Percentage (**A**) and MFI (**B**) of CXCR2^+^ cells within total B cells, total transitional, T1 transitional, T2 transitional, total naive, active naive, resting naive, USM, total DN, DN1, DN2, DN3, SM B cells and ASCs in HC (*n* = 47) and SCI patients (*n* = 51) at the acute (0-4dpi; *n* = 31), subacute/intermediate (16-46dpi; *n* = 37) and chronic (189-379dpi; *n* = 27) phases post-SCI. Mean (± SD) is depicted. Differences in SCI patients over time were analyzed using linear mixed-effects models and post-hoc Tukey HSD tests, and depicted in the figure using asterisks. SCI measurements at each time point were compared with HC (reference group) using a Steel test for nonparametric multiple comparisons, and depicted in the figure using hash marks. *^/#^*p* < 0.05, **^/##^*p* < 0.01, ***^/###^*p* < 0.001, ****^/####^*p* < 0.0001. *ASCs, antibody-secreting cells. CXCR*,* CXC-motif chemokine receptor. DN*,* double negative. Dpi*,* days post-injury. HC*,* healthy controls. Interm.*,* subacute/intermediate. MFI*,* median fluorescence intensity. SCI*,* spinal cord injury. SM*,* switched memory. Trans.*,* transitional. USM*,* unswitched memory.*

CXCR2^+^ cell frequencies were significantly decreased in acute SCI versus HC and increased again over time post-SCI within the total B cell population, as well as in the transitional, naive, USM, DN3 and SM B cell subsets (Fig. 9A, *p*-values in Table S9). For total DN and DN1 B cells, CXCR2^+^ cell frequencies were decreased in the chronic phase post-SCI compared to HC. Differences in CXCR2 surface expression between the study groups were less clear, although the highest expression levels were seen in the acute phase post-SCI within T1, total DN and DN1 B cells (Fig. 9B, *p*-values in Table S9). For several other B cell subsets, such as total naive, resting naive, USM and SM B cells, peak levels of CXCR2 expression were shown in subacute/intermediate SCI.

**Fig. 10:**
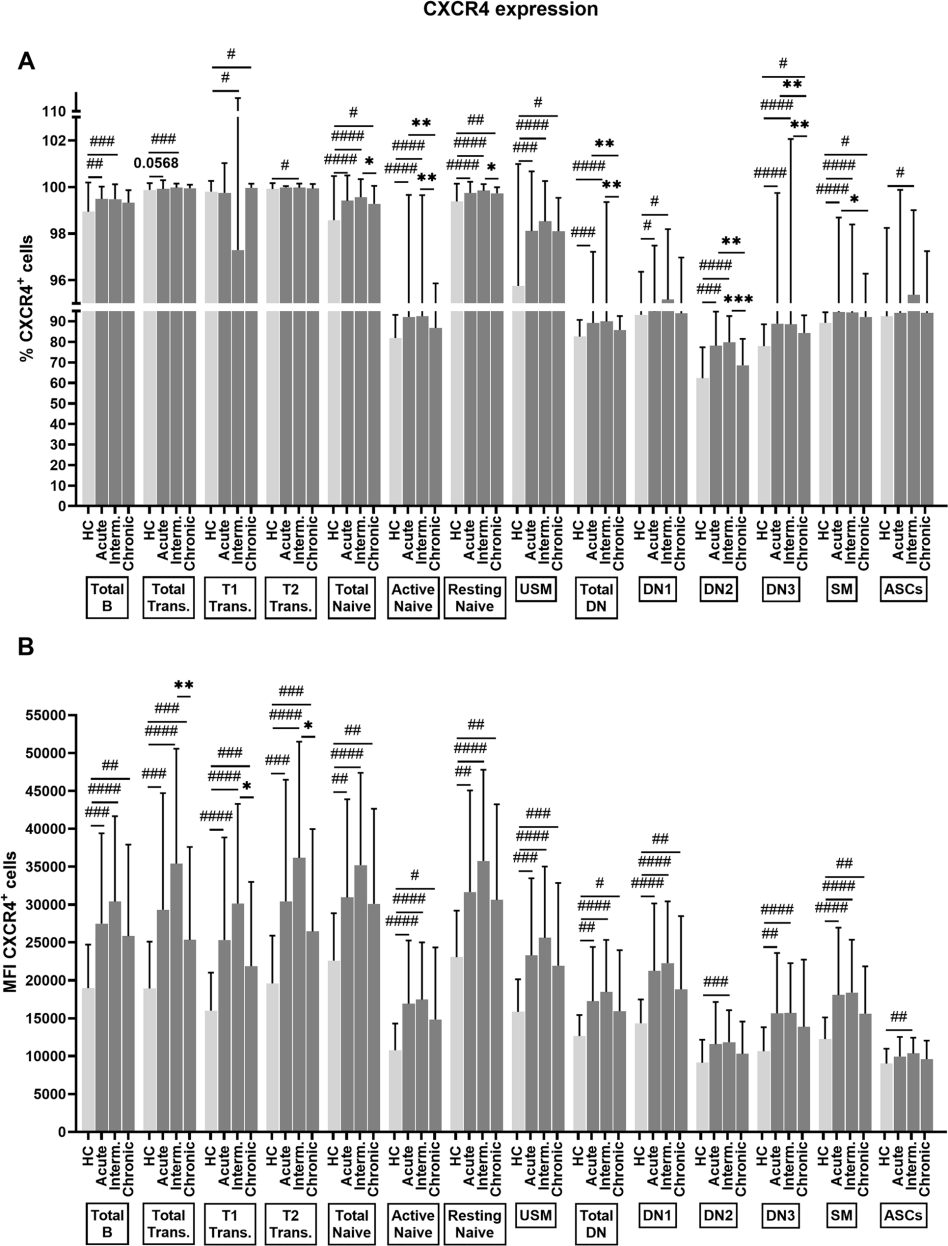
CXCR4 expression on B cell subsets of HC and SCI patients, at different phases post-SCI. Percentage (**A**) and MFI (**B**) of CXCR4^+^ cells within total B cells, total transitional, T1 transitional, T2 transitional, total naive, active naive, resting naive, USM, total DN, DN1, DN2, DN3, SM B cells and ASCs in HC (*n* = 47) and SCI patients (*n* = 51) at the acute (0-4dpi; *n* = 31), subacute/intermediate (16-46dpi; *n* = 37) and chronic (189-379dpi; *n* = 27) phases post-SCI. Mean (± SD) is depicted. Differences in SCI patients over time were analyzed using linear mixed-effects models and post-hoc Tukey HSD tests, and depicted in the figure using asterisks. SCI measurements at each time point were compared with HC (reference group) using a Steel test for nonparametric multiple comparisons, and depicted in the figure using hash marks. *^/#^*p* < 0.05, **^/##^*p* < 0.01, ***^/###^*p* < 0.001, ^*####*^*p* < 0.0001. *ASCs, antibody-secreting cells. CXCR*,* CXC-motif chemokine receptor. DN*,* double negative. Dpi*,* days post-injury. HC*,* healthy controls. Interm.*,* subacute/intermediate. MFI*,* median fluorescence intensity. SCI*,* spinal cord injury. SM*,* switched memory. Trans.*,* transitional. USM*,* unswitched memory.*

Although CXCR4 is highly expressed on B cells, significant changes were evident between the study groups in CXCR4^+^ cells and CXCR4 surface expression within the different B cell subsets (Fig. 10). In general, frequencies of CXCR4^+^ cells were significantly elevated in acute and/or subacute/intermediate SCI when compared with HC for total B cells and all included B cell subsets, and compared to the chronic phase for naive, USM, DN3 and SM B cell subsets (Fig. 10A, *p*-values in Table S10). Only for T1 transitional B cells, we observed significantly decreased CXCR4^+^ cell frequencies in the subacute/intermediate phase compared to HC, potentially due to the large standard deviation at that time point (Fig. 10A). For CXCR4 surface expression, a similar expression pattern was seen for total B cells and all B cell subsets: increased levels of CXCR4 surface expression at all phases in SCI patients versus HC, with a peak in the subacute/intermediate phase post-SCI (Fig. 10B, *p*-values in Table S10).

**Fig. 11:**
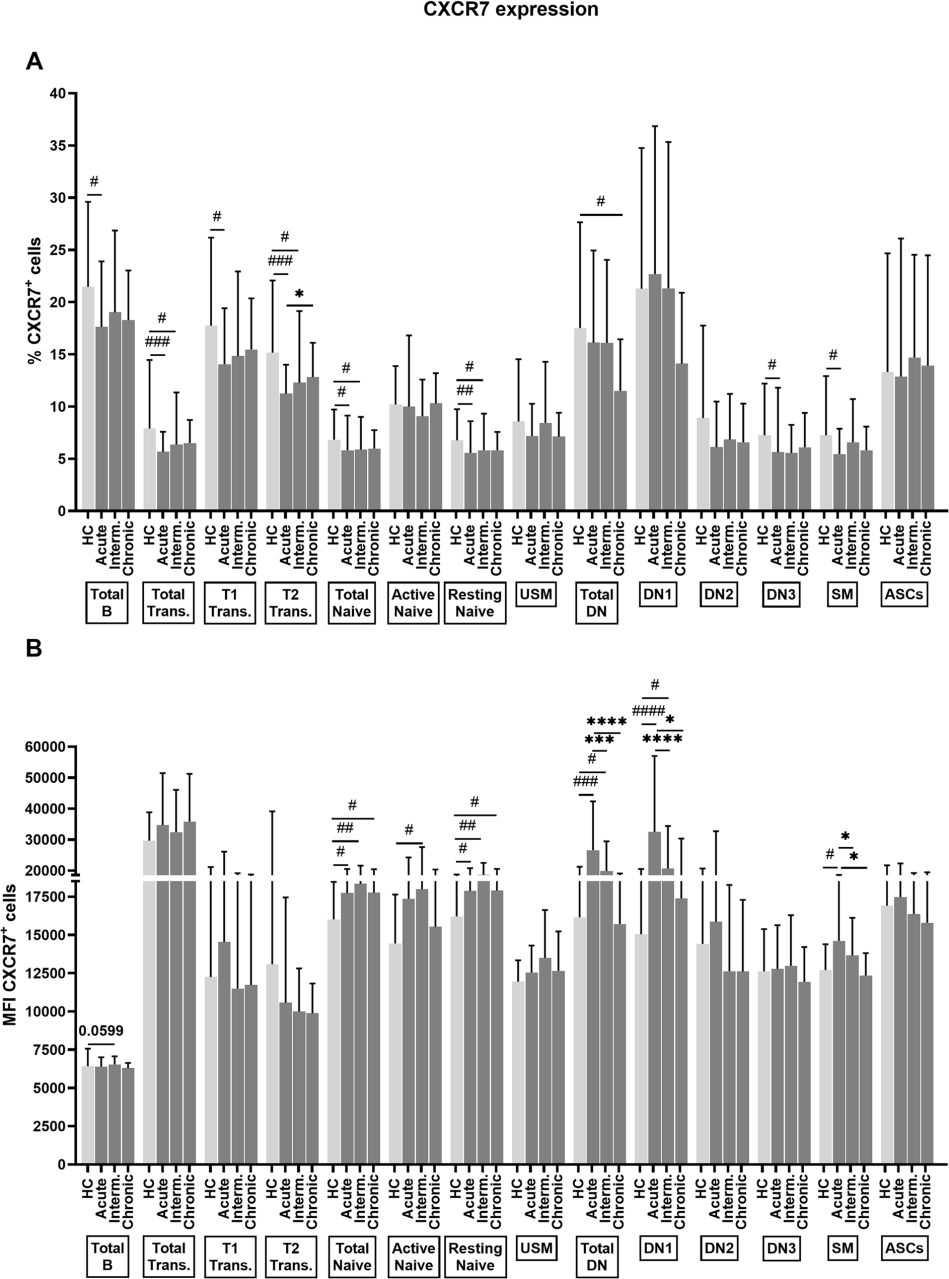
CXCR7 expression on B cell subsets of HC and SCI patients, at different phases post-SCI. Percentage (**A**) and MFI (**B**) of CXCR7^+^ cells within total B cells, total transitional, T1 transitional, T2 transitional, total naive, active naive, resting naive, USM, total DN, DN1, DN2, DN3, SM B cells and ASCs in HC (*n* = 47) and SCI patients (*n* = 51) at the acute (0-4dpi; *n* = 31), subacute/intermediate (16-46dpi; *n* = 37) and chronic (189-379dpi; *n* = 27) phases post-SCI. Mean (± SD) is depicted. Differences in SCI patients over time were analyzed using linear mixed-effects models and post-hoc Tukey HSD tests, and depicted in the figure using asterisks. SCI measurements at each time point were compared with HC (reference group) using a Steel test for nonparametric multiple comparisons, and depicted in the figure using hash marks. *^/#^*p* < 0.05, ^*##*^*p* < 0.01, ***^/###^*p* < 0.001, ****^/####^*p* < 0.0001. *ASCs, antibody-secreting cells. CXCR*,* CXC-motif chemokine receptor. DN*,* double negative. Dpi*,* days post-injury. HC*,* healthy controls. Interm.*,* subacute/intermediate. MFI*,* median fluorescence intensity. SCI*,* spinal cord injury. SM*,* switched memory. Trans.*,* transitional. USM*,* unswitched memory.*

Similar to CXCR2, the frequencies of CXCR7^+^ total B cells and B cell subsets tended to be decreased in SCI patients compared to HC, with the lowest levels in the acute phase (Fig. 11A, *p*-values in Table S11). This was observed for total B cells, all transitional subsets, total naive, resting naive, DN3 and SM B cells. For total DN B cells, the lowest frequency of CXCR7^+^ cells was present in chronic SCI, which was significantly lower compared to HC (*p* = 0.0218) (Fig. 11A). In contrast, CXCR7 surface expression was increased on B cell subsets of SCI patients compared to HC, with peak levels in acute SCI for total DN, DN1 and SM B cells, and in the subacute/intermediate phase for all naive B cell subsets (Fig. 11B, *p*-values in Table S11).

In summary, the MIF receptors CD74, CD44 and CXCR4 were upregulated on B cell subsets post-SCI, with most pronounced increases for CD44. Furthermore, although CXCR2^+^ and CXCR7^+^ cells were downregulated in SCI patients, their surface expression was upregulated in SCI B cells. This indicates that, although there is an acute immune cell reduction, the complete MIF/CD74 axis is upregulated on B cells post-SCI, suggesting that the MIF/CD74 axis plays an important role in post-SCI B cell responses.

### Blocking the MIF/CD74 axis negatively affects B cell proliferation, activation and inflammatory cytokine production

**Fig. 12:**
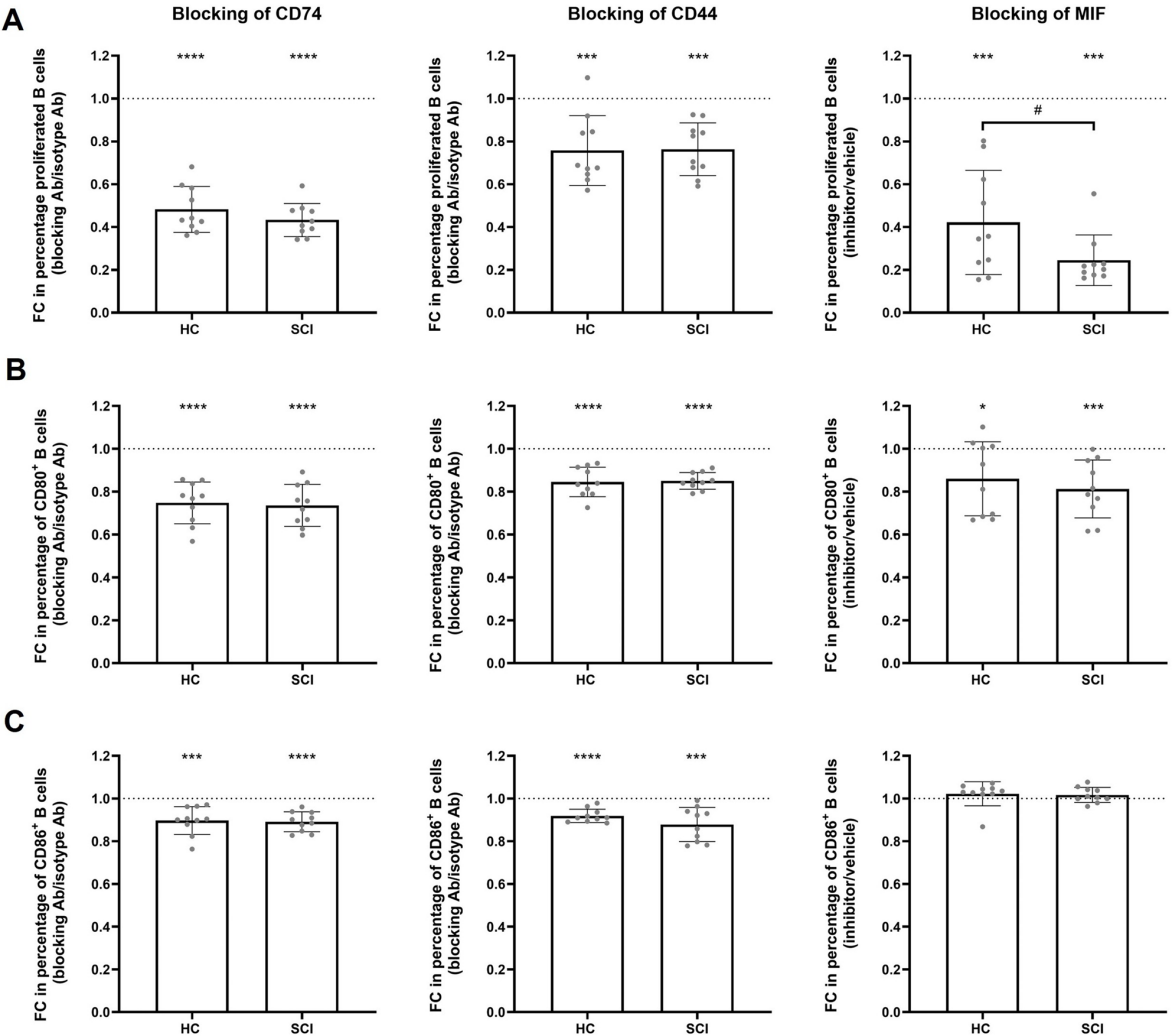
Effects of MIF/CD74 axis blocking on B cell proliferation and activation. Fold change in the percentage of proliferating (**A**), CD80^+^ (**B**) and CD86^+^ (**C**) B cells after blocking of CD74, CD44 or MIF for 72 h in B cell cultures from HC (*n* = 10) and SCI patients (*n* = 10). Mean (± SD) is depicted. Dashed line depicts the control condition. Differences between the blocked and control conditions were analyzed using one-tailed t-tests or Wilcoxon signed-rank tests, and depicted in the figure using asterisks. Differences between HC and SCI patients were analyzed using one-tailed t-tests or Mann-Whitney tests, and depicted in the figure using hash marks. *^/#^*p* < 0.05, ***p < 0.001, ****p < 0.0001. *Ab, antibody. FC*,* fold change. HC*,* healthy controls. MIF*,* macrophage migration inhibitory factor.*

Binding of MIF to its cell surface receptors induces cell proliferation and inflammatory cytokine production [24]. Since MIF plasma levels and MIF receptor expression on B cells were increased post-SCI, we aimed to study the potential functional effects of increased MIF/CD74 activation on B cells of SCI patients. For this, we performed *in vitro* functional assays to assess B cell proliferation (CFSE), activation (CD80, CD86) and cytokine production (IL-1β, TNF-α, IL-6, IL-10) after blocking of CD74, CD44 or MIF for 72 h. As shown by the fold change in comparison to an isotype control antibody (CD74, CD44) or vehicle (MIF), inhibition of CD74, CD44 or MIF significantly reduced B cell proliferation in SCI patients (CD74: *p* < 0.0001, CD44/MIF: *p* = 0.0002) and HC (CD74: *p* < 0.0001, CD44: *p* = 0.0004, MIF: *p* = 0.0005) (Fig. 12A). Interestingly, B cell proliferation was significantly lower after MIF blocking in SCI patients compared to HC (*p* = 0.0376). Additionally, B cell activation was significantly reduced in SCI patients and HC after blocking of the MIF/CD74 axis (Fig. 12B-C). Blocking of CD74, CD44 or MIF significantly reduced frequencies of CD80^+^ B cells in both SCI patients (CD74/CD44: *p* < 0.0001, MIF: *p* = 0.0010) and HC (CD74/CD44: *p* < 0.0001, MIF: *p* = 0.0420) (Fig. 12B). However, only blocking of CD74 and CD44, not MIF, reduced frequencies of CD86^+^ B cells in SCI patients (CD74: *p* < 0.0001, CD44: *p* = 0.0003, MIF: *p* = 0.0833) and HC (CD74: *p* = 0.0003, CD44: *p* < 0.0001, MIF: *p* = 0.1184) (Fig. 12C).

**Fig. 13:**
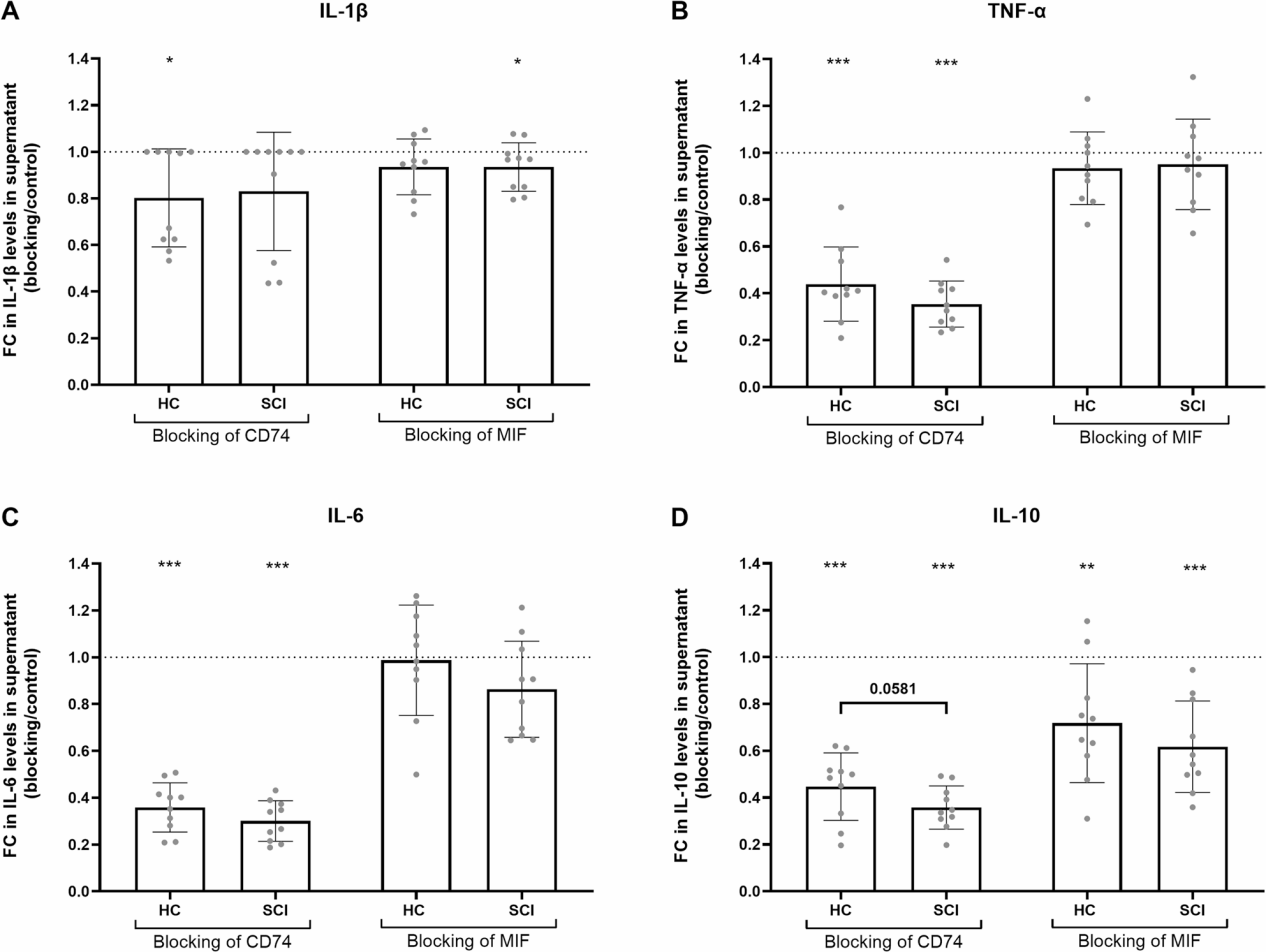
Effects of MIF/CD74 axis blocking on cytokine production by B cells. Fold change in levels of IL-1β (**A**), TNF-α (**B**), IL-6 (**C**) and IL-10 (**D**) after blocking of CD74 or MIF for 72 h in the supernatant of B cell cultures from HC (*n* = 10) and SCI patients (*n* = 10). Mean (± SD) is depicted. Dashed line depicts the control condition. Differences between the blocked and control conditions were analyzed using one-tailed t-tests or Wilcoxon signed-rank tests, and depicted in the figure using asterisks. Differences between HC and SCI patients were analyzed using one-tailed t-tests or Mann-Whitney tests. *p < 0.05, **p < 0.01, ***p < 0.001. *FC, fold change. HC*,* healthy controls. IL*,* interleukin. MIF*,* macrophage migration inhibitory factor. TNF*,* tumor necrosis factor.*

The culture supernatant of these *in vitro* assays was used to analyze the effects of CD74 and MIF blocking on the production of the pro-inflammatory cytokines IL-1β, TNF-α and IL-6, and the anti-inflammatory cytokine IL-10 by B cells using a multiplex immunoassay. CD74 blocking significantly reduced IL-1β expression in HC (*p* = 0.0156), and TNF-α, IL-6 and IL-10 expression in both HC (*p* = 0.0004, *p* = 0.0010 and *p* = 0.0001, respectively) and SCI patients (*p* = 0.0010, *p* = 0.0010 and *p* = 0.0008, respectively) (Fig. 13). Blocking of MIF significantly reduced IL-1β expression in SCI patients (*p* = 0.0336), and IL-10 expression in both HC (*p* = 0.0068) and SCI patients (*p* = 0.0009). Interestingly, CD74 blocking induced a trend toward reduced IL-10 expression in SCI patients compared to HC (*p* = 0.0581).

Thus, blocking of the MIF/CD74 axis in primary B cells decreased B cell function, as we observed reduced proliferation, activation and cytokine production. Here, the effects tended to be more pronounced in B cells from SCI patients, which implies that blocking of the MIF/CD74 axis could be a relevant target to interfere with post-SCI B cell responses.

## Discussion

In this study, we characterized the entire MIF/CD74 axis across the immune system and analyzed its impact on B cell function after SCI. Our findings showed that MIF plasma levels were increased in SCI patients. Decreased intracellular MIF expression within circulating immune cells suggested that the spinal cord is the main source of these upregulated MIF levels post-SCI. Additionally, an early immune cell reduction was triggered post-SCI, indicated by the decreased absolute numbers of total and MIF/CD74 axis-expressing immune cells in SCI patients. In contrast, MIF receptor surface expression was upregulated and frequencies of CD74^+^, CD44^+^ and CXCR4^+^ B cell subsets were increased during the subacute/intermediate phase post-SCI, which indicates the involvement of the complete MIF/CD74 axis in post-SCI B cell responses. Importantly, decreased B cell proliferation, activation and cytokine production were indicated after blocking of not only MIF, but also CD74 and CD44, in primary B cell cultures of SCI patients and HC. The effects of *in vitro* interference with MIF/CD74 axis signaling were more evident in B cells from SCI patients, indicating its potential as a therapeutic intervention for traumatic SCI in the future.

Our study indicated a significant increase in MIF plasma levels in patients with traumatic SCI compared to HC. This upregulation suggests that MIF can influence B cell responses throughout all the different phases following the injury. Previously, we did not find differences in MIF plasma levels between HC and SCI patients at ≤ 1 month and > 1 month post-injury, which could be due to the small sample size of the patient cohort [38]. Although we did not observe significant increases when analyzing the different time points separately, MIF plasma levels were the highest in the acute and chronic phases post-SCI. This corresponds with prior studies that reported increased MIF levels in acute and chronic SCI patients [21–23]. In another study, unchanged MIF levels were found between subacute SCI patients and HC [41]. This is similar to our patient cohort, in which we observed the lowest MIF plasma levels in the subacute and intermediate phases compared to the other phases post-SCI. Although the average MIF plasma levels were elevated in SCI patients compared to HC, it must be noted that a subset of SCI patients displayed levels comparable to those of HC. Moreover, we did not identify any clinical associations among the SCI patients with higher MIF plasma levels, which could be due to limited availability of detailed clinical information for some patients at some of the time points. The increase in MIF plasma levels post-SCI was not caused by upregulation of MIF in circulating immune cells, as MIF expression levels in the main immune cell subsets were reduced in SCI patients compared to HC. Thus, we hypothesize that the injured spinal cord is the main source of the increased MIF plasma levels after traumatic SCI. Following the injury, MIF produced within the spinal cord could leak into the circulation via the disrupted blood-spinal cord barrier. This was already shown in traumatic SCI rat models, where increased MIF mRNA and protein expression were detected in injured spinal cord tissue, infiltrated astrocytes and microglia in the acute time point post-SCI [18–20, 28, 42]. Further research using human post-mortem spinal cord tissue could verify these findings in the human context. Furthermore, the highest MIF expression levels were evidenced in T cell subsets and cytokine-producing NK cells, followed by B cells and intermediate monocytes, but it is unclear whether the increased MIF^+^ cell frequencies have any biological implications, as the frequencies were already above 90% for these immune cell subsets.

Our elaborate analysis of all members of the MIF/CD74 axis in a longitudinal SCI patient cohort indicated decreased absolute numbers of immune cells and MIF^+^ immune cells, as well as MIF/CD74 axis-expressing B cells, up to the subacute/intermediate phase post-SCI. This points toward an early, temporary reduction in peripheral immune cells in SCI patients that gradually increases again over time. Other studies have also observed this acute immune cell reduction in SCI patients, that primarily affected the lymphocyte compartment [43–45]. Importantly, the numerical increase in immune cells over time does not necessarily indicate restored immune function, which may remain impaired over the long term. Decreased NK cell cytotoxicity, as well as reduced lymphocyte proliferation, have already been observed following traumatic SCI [46–48]. Furthermore, the interpretation of circulating immune cell numbers might have been influenced by the lack of information about clinical factors inherent to acute SCI management, such as blood loss and blood transfusion. Despite this early reduction in absolute numbers of circulating immune cells, the frequencies of total T cells, total B cells, classical and intermediate monocytes increased post-SCI. This could suggest that these cells are essential players in the immune response post-SCI, as they appear to be the least affected by the immune cell reduction compared to the other immune cell subsets. In a previous study using a smaller SCI cohort, we demonstrated increased circulating CD74^+^ cell frequencies in total, transitional, naive and USM B cells in a smaller cohort of SCI patients, both at ≤ 1 month and > 1 month post-injury, when compared with HC [38]. Additionally, CD74 surface expression was increased on transitional, naive and USM B cells of SCI patients at > 1 month post-injury [38]. In the current study, we did not detect changes in the frequency of CD74^+^ cells nor in CD74 surface expression in total B cells. This could be due to differences in patient characteristics, patient numbers and included time points between our two studies. Nevertheless, upregulation of CD74 on B cell subsets was still evident, including transitional, naive and DN B cells. In contrast, ASCs displayed decreased CD74^+^ cell frequencies and CD74 surface expression in SCI patients compared to HC. A possible explanation could be that ASCs might not require MIF/CD74 axis signaling, as they no longer differentiate or proliferate. Alternatively, the decreased CD74 expression could also be the result of cellular changes that are needed to initiate antibody secretion. Of all B cell subsets, active naive and DN2 B cells displayed the highest CD74 surface expression. Interestingly, it has been shown in systemic lupus erythematosus (SLE) that active naive B cells served as a precursor for DN2 B cells, which differentiated into ASCs that produced autoreactive antibodies [49, 50]. Accordingly, the MIF/CD74 axis could be responsible for the increased ASC frequencies post-SCI by promoting the differentiation of active naive and DN2 B cells into these ASCs. In this regard, the decreased CD74^+^ ASC frequencies might be resulting from the migration of these cells toward the injured spinal cord in response to MIF.

Furthermore, our current study revealed that not only CD74, but rather the complete MIF/CD74 axis is upregulated post-SCI. This corresponds with other immune-associated pathologies, where the MIF/CD74 axis was found to be upregulated [51–53] and was identified as an important communication pathway between B cells and immune cells [51, 54–57] in multiple autoimmune diseases, such as SLE and myasthenia gravis, COVID-19, Alzheimer’s disease and cancer. More specifically, our analyses revealed the upregulation of CD44^+^ and CXCR4^+^ B cell frequencies, but downregulation of CXCR2^+^ and CXCR7^+^ B cell subsets in SCI patients. It remains unclear whether the increased frequencies of CXCR4^+^ B cell subsets translate into biological relevance, as these were already close to 99% in most B cell subsets. Nevertheless, the decreased frequencies of CXCR2^+^ and CXCR7^+^ B cell subsets could be explained by the reduced absolute numbers of CXCR2^+^ and CXCR7^+^ B cells, while the absolute numbers of CD74^+^, CD44^+^ and CXCR4^+^ B cells remained unchanged. Whether this points toward the importance of CXCR2 and CXCR7, or rather CXCR4, in B cell migration to the spinal cord post-SCI remains to be determined. For all MIF receptors, surface expression levels on B cell subsets were increased in SCI patients, indicating their importance for post-SCI B cell responses. The increased CXCR2 and CXCR7 surface expression on circulating B cells post-SCI could be a compensation mechanism for the loss of B cells expressing these MIF receptors. However, the increased CD74^+^, CD44^+^ and CXCR4^+^ B cell frequencies could also suggest their importance in mediating peripheral MIF signaling, while CXCR2 and CXCR7 might be needed to guide the migration of activated B cells toward the injured spinal cord, as evidenced by their decreased circulating cell frequencies.

The functional relevance of the upregulated MIF receptors in B cells post-SCI was demonstrated in our *in vitro* blocking experiments, which highlighted the importance of CD74, CD44 and MIF in B cell functioning. MIF signaling through CD74 induces intracellular pathway activation and subsequent cell survival, proliferation, migration and cytokine production [24]. We confirmed that blocking of CD74, CD44 or MIF indeed reduced B cell proliferation and cytokine production, presumably by affecting the intracellular signaling pathways. Interestingly, the decrease in B cell functioning upon MIF/CD74 axis inhibition tended to be more pronounced in primary B cells of SCI patients compared to HC. This highlights the therapeutic potential of MIF/CD74 axis interference in SCI. Concerning B cell activation, blocking of the MIF/CD74 axis had a greater effect on CD80 expression, as this marker is mostly upregulated around 48 to 72 h post-stimulation, while CD86 is an early activation marker with a peak expression at 24 h post-stimulation [58, 59]. Our data is also in agreement with other studies that showed reduced B cell proliferation after *in vitro* CD74 or MIF inhibition using human primary B cells or a human multiple myeloma cell line [60–62]. Furthermore, the inhibitory effects on both pro- and anti-inflammatory cytokines suggest that the MIF/CD74 axis could carefully modulate the function of B cells in the inflammatory response post-SCI. It has already been demonstrated that inhibition or knockdown of CD74 or MIF reduced TNF-α, IL-1β and IL-6 levels in the culture supernatant of multiple cell types of both human and animal origin [18, 63–66]. For IL-10, decreased frequencies of IL-10^+^ B cells have been detected in the tumor microenvironment after CD74 inhibition in a mouse model for breast cancer, while MIF inhibition increased IL-10 and IL-1β mRNA, but reduced TNF-α, in RAW264.7 macrophages [37, 67, 68].

This study emphasizes that the involvement of the MIF/CD74 axis in the immune response post-SCI, and more particularly in B cell responses, is not straightforward. The peripheral immune landscape, as well as the expression of the MIF/CD74 axis, are greatly affected by the timing post-SCI. Thus, it remains unclear how MIF/CD74 axis inhibition will affect B cells and SCI pathology *in vivo*. We cannot omit the possibility that the timing post-SCI at which the axis is inhibited influences its effects. For example, MIF/CD74 axis inhibition might not be ideal during the acute peripheral immune cell reduction post-SCI, as it could render SCI patients more susceptible to infections by further suppressing the immune system. Therefore, further research is needed to study the *in vivo* involvement of the MIF/CD74 axis in post-SCI B cell responses and must also focus on the appropriate timing of MIF/CD74 axis inhibition.

## Conclusions

This study showed that SCI triggers an early phase of immune cell reduction, characterized by decreased absolute numbers of total and MIF/CD74 axis-expressing immune cells. However, MIF plasma levels were increased in SCI patients and MIF receptor expression on B cell subsets was upregulated, highlighting the importance of the MIF/CD74 axis in post-SCI B cell responses. Reduced MIF expression in circulating immune cells indicated that the spinal cord is the main source of upregulated MIF levels in SCI patients. The functional significance of this MIF/CD74 axis upregulation in B cells post-SCI was confirmed by decreased B cell activation, proliferation and cytokine production following *in vitro* inhibition of CD74, CD44 and MIF in primary B cells of SCI patients. The stronger effects observed in SCI-derived B cells further underscore their heightened dependence on MIF/CD74 axis signaling. Further research is needed to study the efficacy, safety and timing of *in vivo* MIF/CD74 axis inhibition in B cells using animal models, as well as the involvement of MIF-induced B cell responses in spinal cord pathology. Taken together, our findings reveal an important role for the complete MIF/CD74 axis in post-SCI B cell responses. By demonstrating both phenotypic and functional alterations within this axis in SCI patients, our study contributes to understanding how B cell responses are regulated after a traumatic SCI. Our findings warrant further investigation of the MIF/CD74 axis as a potential target for immunomodulatory strategies in SCI treatment.

## Supplementary Information

Supplementary Material.

## Data Availability

The datasets used and/or analyzed during the current study are available from the corresponding author on reasonable request.

## Abbreviations

Ab: Antibody
AIS: American Spinal Injury Association (ASIA) impairment scale
ASCs: Antibody-secreting cells
CD40L: CD40 ligand
CFSE: Carboxyfluorescein succinimidyl ester
CM: Culture medium
CTLs: Cytotoxic T cells
CV: Coefficient of variation
CXCR: CXC-motif chemokine receptor
DCs: Dendritic cells
DN B cells: Double negative B cells
dpi: Days post-injury
ELISA: Enzyme-linked immunosorbent assay
ERK: Extracellular signal-regulated kinase
FBS: Fetal bovine serum
FC: Fold change
FVD: Fixable viability dye
HC: Healthy controls
Ig: Immunoglobulin
IL: Interleukin
Interm.: Subacute/intermediate phase
MAPK: Mitogen-activated protein kinase
MFI: Median fluorescence intensity
MIF: Macrophage migration inhibitory factor
mRNA: Messenger ribonucleic acid
NA: Not available
NF-κB: Nuclear factor κB
NK cells: Natural killer cells
PBMC: Peripheral blood mononuclear cells
PBS: Phosphate-buffered saline
RBCs: Red blood cells
RT: Room temperature
SCI: Traumatic spinal cord injury
SLE: Systemic lupus erythematosus
SM B cells: Switched memory B cells
Th cells: Helper T cells
TNF-α: Tumor necrosis factor-α
Trans.: Transitional
tSNE: t-Distributed Stochastic Neighbor Embedding
USM B cells: Unswitched memory B cells
w: weeks
