# Supplementary material for: Enhanced MIF/CD74 axis activity shapes B cell functioning following traumatic spinal cord injury
Source: J Neuroinflammation. 2026 Apr 22;23:194. doi: 10.1186/s12974-026-03821-3 (PMC13248257; doi:10.1186/s12974-026-03821-3)
Supplement: Supplementary file 1 — Supplementary Material. [file 12974_2026_3821_MOESM1_ESM.docx]

**SUPPLEMENTARY MATERIAL**

**ENHANCED MIF/CD74 AXIS ACTIVITY SHAPES B CELL FUNCTIONING**

**FOLLOWING TRAUMATIC SPINAL CORD INJURY**

*Serina Rubio, Lien Beckers, Hanne Coenen, Charlotte C.M. van Laake-Geelen, Bart Depreitere,*

*Sven Bamps, Erwin M.J. Cornips, Eveleen Buelens, Diedrik Peuskens, Jens Deckers,*

*Veerle Somers and Judith Fraussen.*

**Table S1 – Antibody panel for the analysis of absolute immune cell numbers.**

| **Marker** | **Fluorochrome** | **Company** | **Category number** | **Dilution** |
| --- | --- | --- | --- | --- |
| CD3 | FITC | BioLegend | 300306 | 1/100 |
| CD45 | PerCP-Cy5.5 | BioLegend | 304027 | 1/25 |
| CD19 | BV421 | BioLegend | 302233 | 1/50 |
| CD14 | BV605 | BioLegend | 301834 | 1/25 |
| CD56 | PE-Dazzle594 | BioLegend | 318348 | 1/50 |
| HLA-DR | Alexa Fluor700 | BioLegend | 307626 | 1/50 |

**Table S2 – First set of antibodies for the MIF/CD74 axis screening.**

| **Marker** | **Fluorochrome** | **Company** | **Category number** | **Dilution** |
| --- | --- | --- | --- | --- |
| CD16 | BV480 | BD Biosciences | 566171 | 1/200 |
| CD45RA | BV570 | BioLegend | 304131 | 1/100 |
| CD24 | BV605 | BioLegend | 311123 | 1/20 |
| CD11c | BV711 | BioLegend | 301629 | 1/50 |
| CD14 | PE-Fire700 | BioLegend | 399221 | 1/2000 |
| IgG | Alexa Fluor700 | BD Biosciences | 561296 | 1/20 |
| Viability Dye | Zombie NIR | BioLegend | 423105 | 1/4000 |

**Table S3 – Second set of antibodies for the MIF/CD74 axis screening.**

| **Marker** | **Fluorochrome** | **Company** | **Category number** | **Dilution** |
| --- | --- | --- | --- | --- |
| CD38 | BV421 | BioLegend | 356617 | 1/100 |
| CD3 | Pacific Blue | BioLegend | 300418 | 1/500 |
| IgM | BV650 | BioLegend | 314525 | 1/100 |
| CXCR4 (CD184) | BV785 | BioLegend | 306529 | 1/20 |
| CD44 | FITC | BioLegend | 103021 | 1/500 |
| CD8 | PerCP | BioLegend | 344707 | 1/50 |
| IgA | PerCP-Vio700 | Miltenyi Biotec | 130-117-005 | 1/200 |
| CD27 | RealBlue744 | BD Biosciences | 570713 | 1/50 |
| CD4 | PerCP-Fire806 | BioLegend | 344693 | 1/50 |
| CD74 | PE | BioLegend | 326807 | 1/20 |
| CXCR7 | PE-Dazzle594 | BioLegend | 331117 | 1/20 |
| CD56 (NCAM) | PE-Fire640 | BioLegend | 392431 | 1/200 |
| IgD | PE-Cy5 | BioLegend | 348249 | 1/500 |
| CXCR2 (CD182) | PE-Cy7 | BioLegend | 320715 | 1/20 |
| CD21 | APC-Fire750 | BioLegend | 354919 | 1/20 |
| CD19 | APC-Fire810 | BioLegend | 302271 | 1/250 |


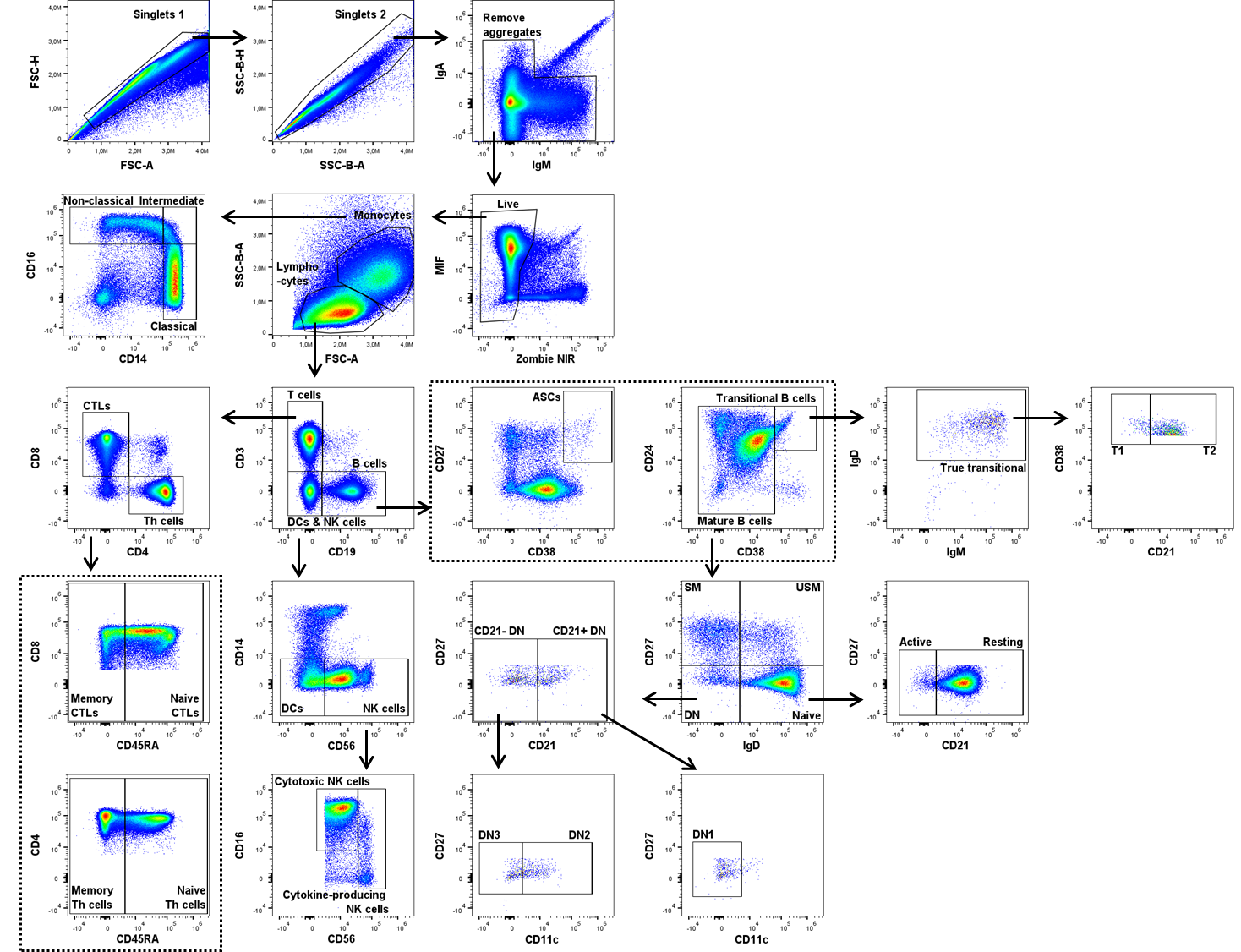


**Figure S1 –** **Representative gating strategy to define the expression of members of the MIF/CD74 axis in immune** **cell subsets.** The gating strategy for one representative HC is shown. Single cells were selected based on FSC‑A and FSC-H, followed by SSC-B-A and SSC-B-H. Next, aggregates were removed based on IgM BV605 and IgA PerCP-Vio700 antibodies, and living cells were gated using a Zombie NIR dye and MIF AF647 antibody. Lymphocytes and monocytes were selected using FSC-A and SSC-B-A. Within the monocyte population, classical, intermediate and non-classical monocytes were gated based on CD16 and CD14 expression. T cells (CD3^+^), B cells (CD19^+^) and DCs & NK cells (CD3^-^CD19^-^) were gated from the lymphocytes. CD3^+^ T cells were further divided into CD4^+^ Th cells and CD8^+^ CTLs, and subsequently divided into memory (CD45RA^-^) and naive (CD45RA^+^) cells. DCs and NK cells were differentiated by CD14 and CD56, and NK cells were further classified as cytotoxic (CD16^+^) or cytokine-producing (CD16^-/+^) NK cells. CD19^+^ B cells were further divided into ASCs, transitional and mature B cells based on CD24, CD27 and CD38 expression. Transitional B cells were further divided into T1 (CD21^-^) and T2 (CD21^+^) subsets. Mature B cells were divided into SM, USM, DN and naive B cells based on IgD and CD27 expression. Within naive B cells, active (CD21^-^) and resting (CD21^+^) cells were defined. Within DN B cells, DN1 (CD21^+^CD11c^-^), DN2 (CD21^-^CD11c^+^) and DN3 (CD21^-^CD11c^-^) subsets were defined based on CD21 and CD11c expression. Within ASCs, SM, USM, DN and naive B cells, expression of IgA, IgG and/or IgM was defined (not shown). Gating was based on FMO controls for several markers. ASCs, antibody-secreting cells. CTLs, cytotoxic T cells. DCs, dendritic cells. DN, double negative. FMO, fluorescence minus one. FSC-A/H, forward scatter area/height. HC, healthy control. Ig, immunoglobulin. MIF, macrophage migration inhibitory factor. NK cells, natural killer cells. SM, switched memory. SSC-B-A/H, side scatter area/height. Th cells, helper T cells. USM, unswitched memory.

**Table S4 – Blocking antibodies and small molecule inhibitor for the in vitro blocking assays.**

| **Specificity** | **Name** | **Clone** | **Type** | **Company** | **Cat. No.** | **Concentration** |
| --- | --- | --- | --- | --- | --- | --- |
| CD74 blocking | Milatuzumab | / | Humanized IgG1κ | MedChem-Express | HY-P99731 | 10 µg/ml |
|  | Isotype control | MG1-45 | Ultra-LEAF mouse IgG1κ | BioLegend | 401407 | 10 µg/ml |
| CD44 blocking | Anti-CD44 antibody | Hermes-1 | Rat IgG2a | ThermoFisher | MA4400 | 40 µg/ml |
|  | Isotype control | RTK2758 | Ultra-LEAF rat IgG2a | BioLegend | 400543 | 40 µg/ml |
| MIF blocking | ISO1 | / | Small molecule inhibitor | R&D systems | 4288 | 1000 µM |
|  | Vehicle control | / | DMSO | PanReac AppliChem | A3672 | / |

*DMSO, dimethyl sulfoxide.*

**Table S5 – Antibody panel for the in vitro blocking assays.**

| **Marker** | **Fluorochrome** | **Company** | **Category number** | **Dilution** |
| --- | --- | --- | --- | --- |
| CD80 | BV421 | BioLegend | 305222 | 1/20 |
| CD19 | BV650 | BioLegend | 302238 | 1/50 |
| CD86 | BV785 | BioLegend | 305442 | 1/50 |


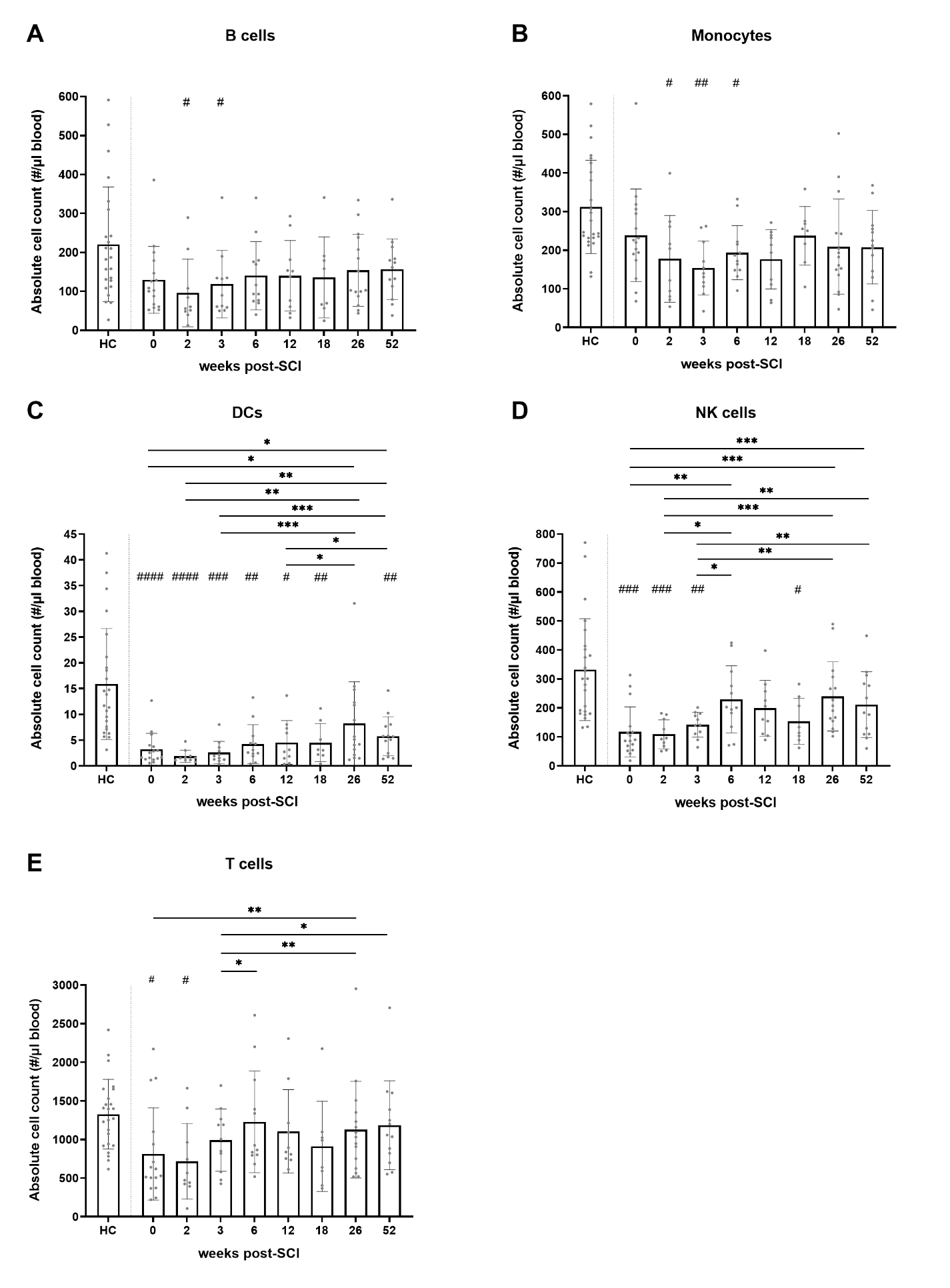


**Figure S2 –** **Absolute numbers of the major immune cell subsets in HC and SCI patients.** Absolute numbers of B cells (**A**), monocytes (**B**), DCs (**C**), NK cells (**D**) and T cells (**E**) in HC (n = 23) and SCI patients (n = 27) at 0 (1-4dpi; n = 16), 2 (15-19dpi; n = 10), 3 (22-34dpi; n = 10), 6 (43-51dpi; n = 11), 12 (80-97dpi; n = 9), 18 (120-134dpi; n = 8), 26 (178-189dpi; n = 14), and 52 (362-396dpi; n = 12) weeks post-SCI. Mean (± SD) is depicted. Differences in SCI patients over time were analyzed using linear mixed-effects models and post-hoc Tukey HSD tests, and depicted in the figure using asterisks. SCI measurements at each time point were compared with HC (reference group) using a Steel test for nonparametric multiple comparisons, and depicted in the figure using hash marks. **^/#^p<0.05, **^/##^p<0.01, ***^/###^p<0.001, ^####^p<0.0001. DCs, dendritic cells. Dpi, days post-injury. HC, healthy controls. NK cells, natural killer cells. SCI, spinal cord injury.*


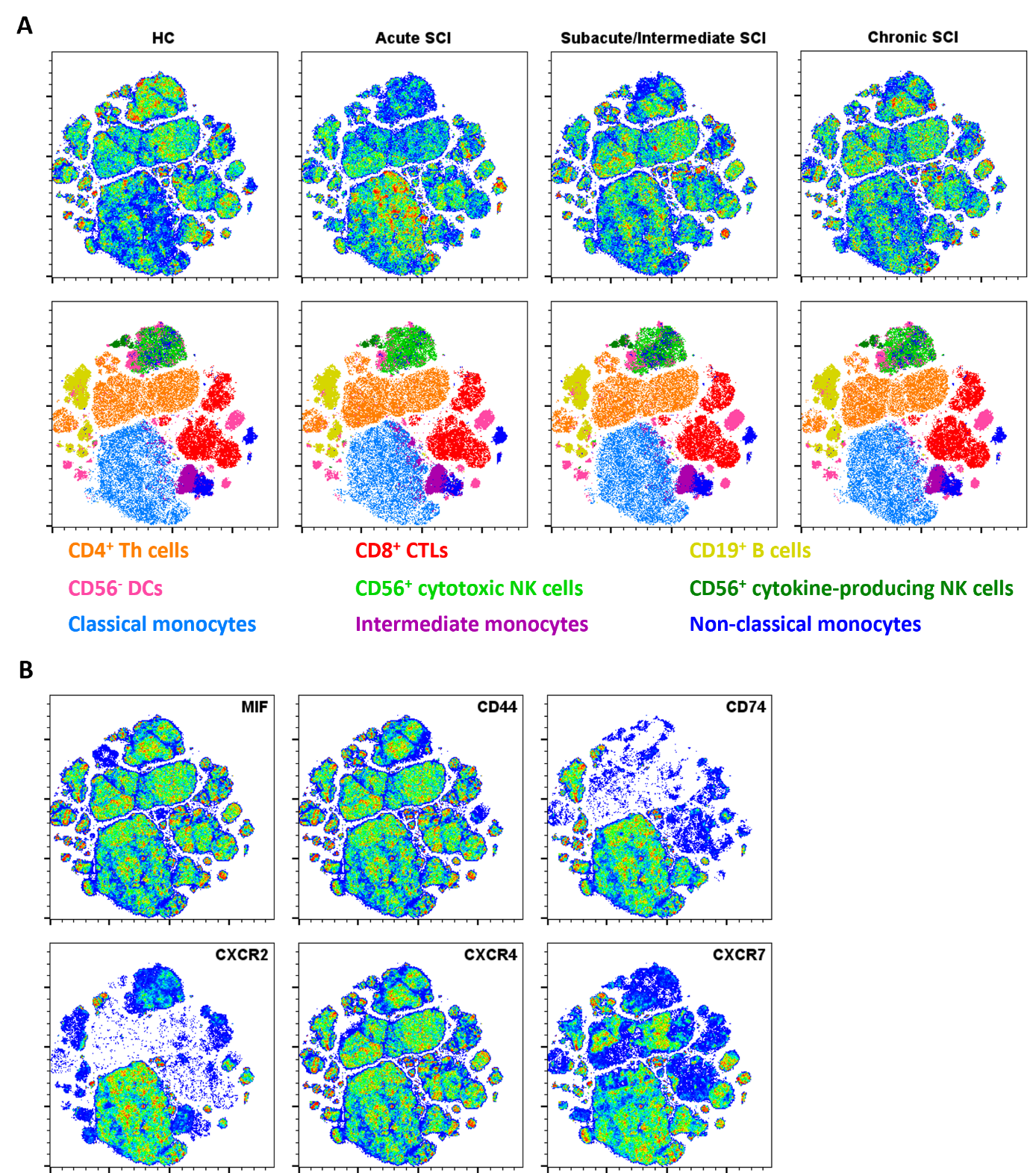


**Figure S3 –** **High-dimensional flow cytometry of the major immune cell subsets and MIF/CD74 axis in HC and SCI patients.** (**A**) tSNE maps showing the major immune cell subsets within single live cells of HC (n = 47) and SCI patients (n = 51) at the acute (0-4dpi; n = 31), subacute/intermediate (16-46dpi; n = 37) and chronic (189-379dpi; n = 27) phases post-SCI. The upper row shows the tSNE maps on pseudocolor plots, while the lower row shows the manually determined gates on the tSNE maps. (**B**) tSNE maps (pseudocolor plots) showing the expression of MIF, CD44, CD74, CXCR2, CXCR4 and CXCR7 within single live cells (n = 98). DCs, dendritic cells. Dpi, days post-injury. HC, healthy control. NK cells, natural killer cells. SCI, spinal cord injury. tSNE, t-Distributed Stochastic Neighbor Embedding.


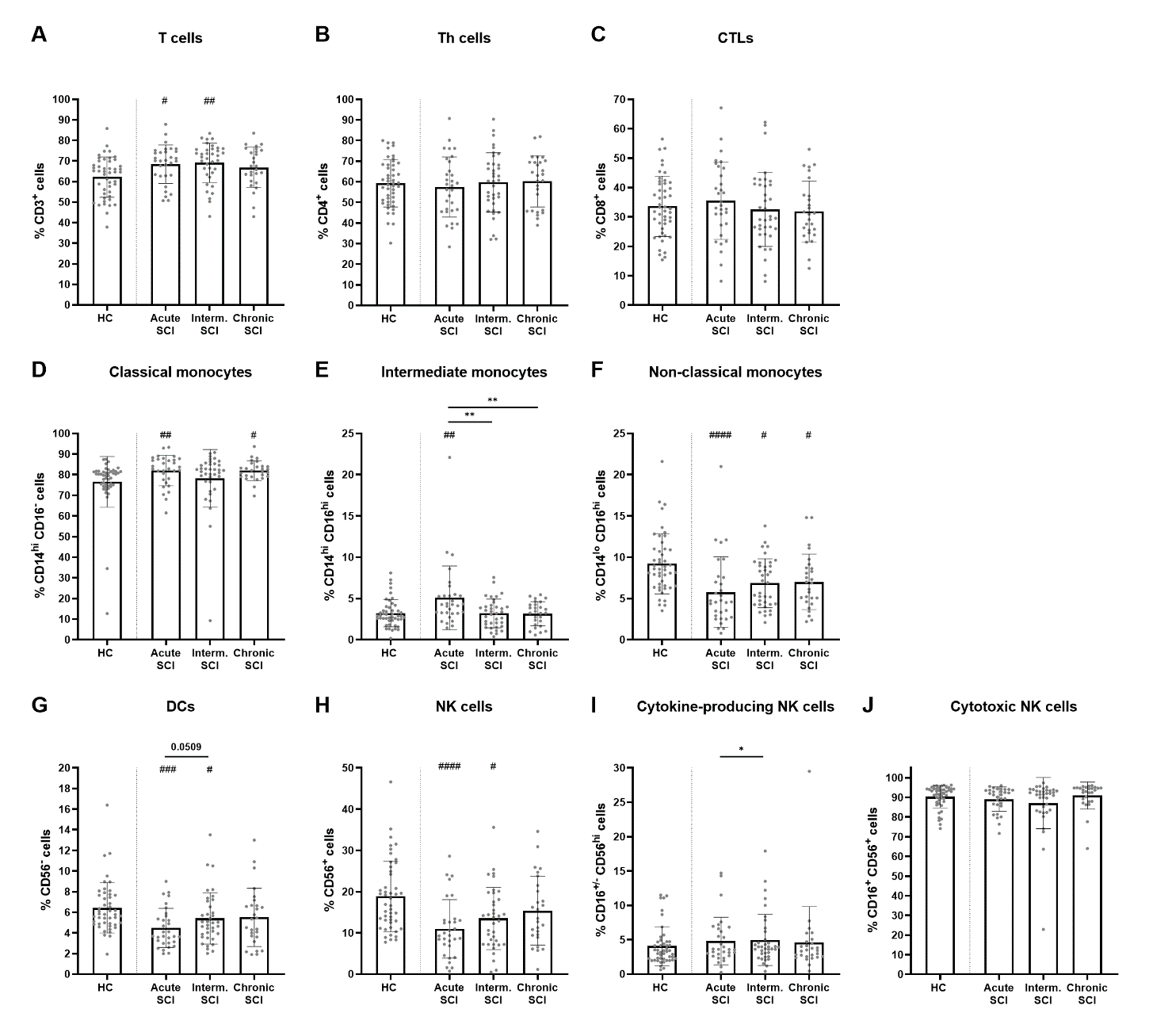


**Figure S4 –** **Percentage of immune cell subsets in HC and SCI patients, at different phases post-SCI.** Percentage of T cells (**A**), including Th cells (**B**) and CTLs (**C**), classical monocytes (**D**), intermediate monocytes (**E**), non-classical monocytes (**F**), DCs (**G**), NK cells (**H**), including cytokine-producing (**I**) and cytotoxic (**J**) NK cells, in HC (n = 47) and SCI patients (n = 51) at the acute (0-4dpi; n = 31), subacute/intermediate (16-46dpi; n = 37) and chronic (189-379dpi; n = 27) phases post-SCI. Mean (± SD) is depicted. Differences in SCI patients over time were analyzed using linear mixed-effects models and post-hoc Tukey HSD tests, and depicted in the figure using asterisks. SCI measurements at each time point were compared with HC (reference group) using a Steel test for nonparametric multiple comparisons, and depicted in the figure using hash marks. *^/#^p<0.05, **^/##^p<0.01, ^###^p<0.001, ^####^p<0.0001. CTLs, cytotoxic T cells. DCs, dendritic cells. Dpi, days post-injury. HC, healthy controls. Interm., subacute/intermediate. NK cells, natural killer cells. Th cells, helper T cells. SCI, spinal cord injury.

**Table S6 – Statistical significance of MIF expression in B cell and major immune cell subsets.**

| **Immune cell subset** | **Time points** | **p-value** |
| --- | --- | --- |
| **B cells** | | |
| Total B | HC vs acute | *MFI: p=0.0197* |
| Total transitional | HC vs acute | *MFI: p=0.0319* |
| *T2* | HC vs acute | *MFI: p=0.0225* |
| Total naive | HC vs acute | *MFI: p=0.0387* |
| *Resting naive* | HC vs acute | *MFI: p=0.0323* |
| USM | HC vs acute | *MFI: p=0.0061* |
| Total DN | HC vs acute | *MFI: p=0.0415* |
| *DN2* | HC vs acute | *MFI: p=0.0566* |
| SM | HC vs acute | *MFI: p=0.0056* |
| ASCs | HC vs acute | *MFI: p=0.0006* |
|  | HC vs subacute/intermediate | *MFI: p<0.0001* |
| **Other immune cells** | | |
| DCs | HC vs acute | *%: p=0.0318, MFI: p=0.0058* |
|  | HC vs subacute/intermediate | *MFI: p=0.0134* |
|  | HC vs chronic | *MFI: p=0.0179* |
|  | Acute vs subacute/intermediate | *%: p=0.0145* |
|  | Acute vs chronic | *%: p=0.0095* |
| Cytokine-producing NK cells | HC vs acute | *%: p=0.0055, MFI: p=0.0029* |
|  | Acute vs subacute/intermediate | *MFI: p=0.0077* |
| Non-classical monocytes | HC vs acute | *%: p<0.0001, MFI: p=0.0032* |
|  | HC vs chronic | *MFI: p=0.0319* |
|  | Acute vs subacute/intermediate | *MFI: p=0.0183* |

*DN, double negative. HC, healthy controls. MFI, median fluorescence intensity. MIF, macrophage migration inhibitory factor. SCI, spinal cord injury. SM, switched memory. USM, unswitched memory.*

**Table S7 – Statistical significance of CD74 expression on B cell subsets.**

| **B cell subset** | **Time points** | **p-value** |
| --- | --- | --- |
| Total transitional | HC vs subacute/intermediate | *MFI: p=0.0465* |
| *T2* | HC vs subacute/intermediate | *MFI: p=0.0315* |
| Resting naive | HC vs subacute/intermediate | *%: p=0.0533, MFI: p=0.0476* |
| USM | HC vs subacute/intermediate | *%: p=0.0550* |
| Total DN | HC vs chronic | *%: p=0.0344* |
| ASCs | HC vs subacute/intermediate | *%: p<0.0001, MFI: p=0.0032* |
|  | Acute vs subacute/intermediate | *%: p=0.0385* |
|  | Subacute/intermediate vs chronic | *%: p=0.0047, MFI: p=0.0495* |

*ASCs, antibody-secreting cells. DN, double negative. HC, healthy controls. MFI, median fluorescence intensity. SCI, spinal cord injury. USM, unswitched memory.*

**Table S8 – Statistical significance of CD44 expression on B cell subsets.**

| **B cell subset** | **Time points** | **p-value** |
| --- | --- | --- |
| Total B | HC vs acute | *%: p=0.0562* |
|  | HC vs subacute/intermediate | *%: p=0.0068* |
|  | HC vs chronic | *%: p=0.0247* |
| Total transitional | HC vs subacute/intermediate | *%: p=0.0003, MFI: p=0.0034* |
|  | Acute vs subacute/intermediate | *MFI: p<0.0001* |
|  | Subacute/intermediate vs chronic | *MFI: p<0.0001* |
| *T1* | HC vs acute | *%: p=0.0012* |
|  | HC vs subacute/intermediate | *%: p=0.0010, MFI: p=0.0181* |
|  | Subacute/intermediate vs chronic | *MFI: p=0.0083* |
| *T2* | HC vs subacute/intermediate | *%: p=0.0011, MFI: p=0.0010* |
|  | Acute vs subacute/intermediate | *MFI: p<0.0001* |
|  | Subacute/intermediate vs chronic | *MFI: p<0.0001* |
| Total naive | HC vs subacute/intermediate | *%: p=0.0178* |
|  | HC vs chronic | *%: p=0.0175* |
| *Active naive* | HC vs acute | *%: p=0.0409* |
| *Resting naive* | HC vs subacute/intermediate | *%: p=0.0198* |
|  | HC vs chronic | *%: p=0.0203* |
| USM | HC vs chronic | *MFI: p=0.0595* |
| Total DN | HC vs chronic | *%: p=0.0170* |
| *DN1* | HC vs chronic | *%: p=0.0127, MFI: p=0.0236* |
| *DN3* | HC vs chronic | *MFI: p=0.0401* |

*DN, double negative. HC, healthy controls. MFI, median fluorescence intensity. SCI, spinal cord injury. SM, switched memory. USM, unswitched memory.*

**Table S9 – Statistical significance of CXCR2 expression on B cell subsets.**

| **B cell subset** | **Time points** | **p-value** |
| --- | --- | --- |
| Total B | HC vs acute | *%: p=0.0012* |
|  | HC vs subacute/intermediate | *%:* *p=0.0070* |
|  | Acute vs chronic | *%: p<0.0001* |
|  | Subacute/intermediate vs chronic | *%: p=0.0013* |
| Total transitional | HC vs acute | *%: p=0.0019* |
|  | HC vs subacute/intermediate | *%: p=0.0058* |
| *T1* | HC vs acute | *MFI: p=0.0083* |
| *T2* | HC vs acute | *%: p=0.0055* |
|  | HC vs subacute/intermediate | *%: p=0.0307* |
|  | Acute vs chronic | *%: p=0.0326* |
| Total naive | HC vs acute | *%: p=0.0002, MFI: p=0.0111* |
|  | HC vs subacute/intermediate | *%: p=0.0030, MFI: p<0.0001* |
|  | Acute vs chronic | *%: p<0.0001* |
|  | Subacute/intermediate vs chronic | *%: p=0.0007* |
| *Resting naive* | HC vs acute | *%: p<0.0001, MFI: p=0.0235* |
|  | HC vs subacute/intermediate | *%: p=0.0011, MFI: p=0.0001* |
|  | Acute vs chronic | *%: p<0.0001* |
|  | Subacute/intermediate vs chronic | *%: p=0.0014* |
| USM | HC vs acute | *%: p=0.0007* |
|  | HC vs subacute/intermediate | *%: p=0.0070, MFI: p=0.0229* |
|  | Acute vs chronic | *%: p=0.0084* |
|  | Subacute/intermediate vs chronic | *%: p=0.0452* |
| Total DN | HC vs acute | *MFI: p=0.0013* |
|  | HC vs subacute/intermediate | *MFI: p=0.0082* |
|  | HC vs chronic | *%: p=0.0105* |
|  | Acute vs subacute/intermediate | *MFI: p=0.0056* |
| *DN1* | HC vs acute | *MFI: p<0.0001* |
|  | HC vs subacute/intermediate | *MFI: p=0.0019* |
|  | HC vs chronic | *%: p=0.0072* |
|  | Acute vs subacute/intermediate | *MFI: p=0.0001* |
|  | Acute vs chronic | *%: p=0.0176, MFI: p=0.0028* |
| *DN3* | HC vs acute | *%: p=0.0143* |
| SM | HC vs acute | *%: p=0.0015, MFI: p=0.0280* |
|  | HC vs subacute/intermediate | *%: p=0.0348, MFI: p=0.0058* |
|  | Subacute/intermediate vs chronic | *MFI: p=0.0060* |

*CXCR, CXC-motif chemokine receptor. DN, double negative. HC, healthy controls. MFI, median fluorescence intensity. SCI, spinal cord injury. SM, switched memory. USM, unswitched memory.*

**Table S10 – Statistical significance of CXCR4 expression on B cell subsets.**

| **B cell subset** | **Time points** | **p-value** |
| --- | --- | --- |
| Total B | HC vs acute | *%: p=0.0030, MFI: p=0.0002* |
|  | HC vs subacute/intermediate | *%: p=0.0007, MFI: p<0.0001* |
|  | HC vs chronic | *MFI: p=0.0011* |
| Total transitional | HC vs acute | *%: p=0.0568, MFI: p=0.0001* |
|  | HC vs subacute/intermediate | *%: p=0.0006, MFI: p<0.0001* |
|  | HC vs chronic | *MFI: p=0.0008* |
|  | Subacute/intermediate vs chronic | *MFI: p=0.0069* |
| *T1* | HC vs acute | *MFI: p<0.0001* |
|  | HC vs subacute/intermediate | *%: p=0.0103, MFI: p<0.0001* |
|  | HC vs chronic | *%: p=0.0418, MFI: p=0.0008* |
|  | Subacute/intermediate vs chronic | *MFI: p=0.0158* |
| *T2* | HC vs acute | *MFI: p=0.0001* |
|  | HC vs subacute/intermediate | *%: p=0.0163, MFI: p<0.0001* |
|  | HC vs chronic | *MFI: p=0.0006* |
|  | Subacute/intermediate vs chronic | *MFI: p=0.0111* |
| Total naive | HC vs acute | *%: p<0.0001, MFI: p=0.0028* |
|  | HC vs subacute/intermediate | *% - MFI: p<0.0001* |
|  | HC vs chronic | *%: p=0.0129, MFI: p=0.0010* |
|  | Subacute/intermediate vs chronic | *%: p=0.0148* |
| *Active naive* | HC vs acute | *% - MFI: p<0.0001* |
|  | HC vs subacute/intermediate | *% - MFI: p<0.0001* |
|  | HC vs chronic | *MFI: p=0.0111* |
|  | Acute vs chronic | *%: p=0.0053* |
|  | Subacute/intermediate vs chronic | *%: p=0.0013* |
| *Resting naive* | HC vs acute | *%: p<0.0001,* *MFI: p=0.0027* |
|  | HC vs subacute/intermediate | *% - MFI: p<0.0001* |
|  | HC vs chronic | *%: p=0.0023, MFI: p=0.0013* |
|  | Subacute/intermediate vs chronic | *%: p=0.0242* |
| USM | HC vs acute | *%: p=0.0003, MFI: p=0.0002* |
|  | HC vs subacute/intermediate | *% - MFI: p<0.0001* |
|  | HC vs chronic | *%: p=0.0187, MFI: p=0.0003* |
| Total DN | HC vs acute | *%: p=0.0002, MFI: p=0.0010* |
|  | HC vs subacute/intermediate | *% - MFI: p<0.0001* |
|  | HC vs chronic | *MFI: p=0.0295* |
|  | Acute vs chronic | *%: p=0.0086* |
|  | Subacute/intermediate vs chronic | *%: p=0.0028* |
| *DN1* | HC vs acute | *%: p=0.0403, MFI: p<0.0001* |
|  | HC vs subacute/intermediate | *%: p=0.0128, MFI: p<0.0001* |
|  | HC vs chronic | *MFI: p=0.0051* |
| *DN2* | HC vs acute | *%: p=0.0002* |
|  | HC vs subacute/intermediate | *%: p<0.0001, MFI: p=0.0007* |
|  | Acute vs chronic | *%: p=0.0039* |
|  | Subacute/intermediate vs chronic | *%: p=0.0003* |
| *DN3* | HC vs acute | *%: p<0.0001, MFI: p=0.0020* |
|  | HC vs subacute/intermediate | *% - MFI: p<0.0001* |
|  | HC vs chronic | *%: p=0.0377* |
|  | Acute vs chronic | *%: p=0.0016* |
|  | Subacute/intermediate vs chronic | *%: p=0.0032* |
| SM | HC vs acute | *% - MFI: p<0.0001* |
|  | HC vs subacute/intermediate | *% - MFI: p<0.0001* |
|  | HC vs chronic | *%: p=0.0407, MFI: p=0.0035* |
|  | Acute vs chronic | *%: p=0.0388* |
| ASCs | HC vs subacute/intermediate | *%: p=0.0319, MFI: p=0.0044* |

*ASCs, antibody-secreting cells. CXCR, CXC-motif chemokine receptor. DN, double negative. HC, healthy controls. MFI, median fluorescence intensity. SCI, spinal cord injury. SM, switched memory. USM, unswitched memory.*

**Table S11 – Statistical significance of CXCR7 expression on B cell subsets.**

| **B cell subset** | **Time points** | **p-value** |
| --- | --- | --- |
| Total B | HC vs acute | *%: p=0.0444* |
|  | HC vs subacute/intermediate | *MFI: p=0.0599* |
| Total transitional | HC vs acute | *%: p=0.0005* |
|  | HC vs subacute/intermediate | *%: p=0.0169* |
| *T1* | HC vs acute | *%: p=0.0212* |
| *T2* | HC vs acute | *%: p=0.0005* |
|  | HC vs subacute/intermediate | *%: p=0.0100* |
|  | Acute vs chronic | *%: p=0.0394* |
| Total naive | HC vs acute | *%: p=0.0191, MFI: p=0.0188* |
|  | HC vs subacute/intermediate | *%: p=0.0169, MFI: p=0.0016* |
|  | HC vs chronic | *MFI: p=0.0260* |
| *Active naive* | HC vs subacute/intermediate | *MFI: p=0.0311* |
| *Resting naive* | HC vs acute | *%: p=0.0050, MFI: p=0.0188* |
|  | HC vs subacute/intermediate | *%: p=0.0117, MFI: p=0.0027* |
|  | HC vs chronic | *MFI: p=0.0329* |
| Total DN | HC vs acute | *MFI: p=0.0001* |
|  | HC vs subacute/intermediate | *MFI: p=0.0315* |
|  | HC vs chronic | *%: p=0.0218* |
|  | Acute vs subacute/intermediate | *MFI: p=0.0001* |
|  | Acute vs chronic | *MFI: p<0.0001* |
| *DN1* | HC vs acute | *MFI: p<0.0001* |
|  | HC vs subacute/intermediate | *MFI: p=0.0112* |
|  | Acute vs subacute/intermediate | *MFI: p<0.0001* |
|  | Acute vs chronic | *MFI: p=0.0108* |
| *DN3* | HC vs acute | *%: p=0.0421* |
| SM | HC vs acute | *%: p=0.0382, MFI: p=0.0268* |
|  | Acute vs subacute/intermediate | *MFI: p=0.0249* |
|  | Acute vs chronic | *MFI: p=0.0377* |

*CXCR, CXC-motif chemokine receptor. DN, double negative. HC, healthy controls. MFI, median fluorescence intensity. SCI, spinal cord injury. SM, switched memory. USM, unswitched memory.*
